# L-Proline Synthesis Mutants of *Bacillus subtilis* Overcome Osmotic Sensitivity by Genetically Adapting L-Arginine Metabolism

**DOI:** 10.3389/fmicb.2022.908304

**Published:** 2022-06-16

**Authors:** Daniela Stecker, Tamara Hoffmann, Hannes Link, Fabian M. Commichau, Erhard Bremer

**Affiliations:** ^1^Faculty of Biology, Philipps-University Marburg, Marburg, Germany; ^2^SYNMIKRO Research Center, Philipps-University Marburg, Marburg, Germany; ^3^Max Planck Institute for Terrestrial Microbiology, Marburg, Germany; ^4^Insitute of Microbiology and Genetics, Georg-August-University Göttingen, Göttingen, Germany; ^5^Institute for Biotechnology, BTU Cottbus-Senftenberg, Senftenberg, Germany

**Keywords:** osmotic stress, suppressor mutations, metabolomics, gene regulation, compatible solutes, L-proline

## Abstract

The accumulation of the compatible solute L-proline by *Bacillus subtilis via* synthesis is a cornerstone in the cell’s defense against high salinity as the genetic disruption of this biosynthetic process causes osmotic sensitivity. To understand how *B. subtilis* could potentially cope with high osmolarity surroundings without the functioning of its natural osmostress adaptive L-proline biosynthetic route (ProJ-ProA-ProH), we isolated suppressor strains of *proA* mutants under high-salinity growth conditions. These osmostress-tolerant strains carried mutations affecting either the AhrC transcriptional regulator or its operator positioned in front of the *argCJBD-carAB-argF* L-ornithine/L-citrulline/L-arginine biosynthetic operon. Osmostress protection assays, molecular analysis and targeted metabolomics showed that these mutations, in conjunction with regulatory mutations affecting *rocR-rocDEF* expression, connect and re-purpose three different physiological processes: (i) the biosynthetic pathway for L-arginine, (ii) the RocD-dependent degradation route for L-ornithine, and (iii) the last step in L-proline biosynthesis. Hence, osmostress adaptation without a functional ProJ-ProA-ProH route is made possible through a naturally existing, but inefficient, metabolic shunt that allows to substitute the enzyme activity of ProA by feeding the RocD-formed metabolite γ-glutamate-semialdehyde/Δ^1^-pyrroline-5-carboxylate into the biosynthetic route for the compatible solute L-proline. Notably, in one class of mutants, not only substantial L-proline pools but also large pools of L-citrulline were accumulated, a rather uncommon compatible solute in microorganisms. Collectively, our data provide an example of the considerable genetic plasticity and metabolic resourcefulness of *B. subtilis* to cope with everchanging environmental conditions.

## Introduction

Increases in the environmental osmolarity occur frequently in the varied habitats of microorganisms and impose considerable energetic and growth-restricting constrains on bacterial cells (Gunde-Cimerman et al., 2018). The detrimental effects of high osmolarity on cellular physiology are a consequence of the different osmotic potentials of the cell’s crowded cytoplasm and its surroundings, and of the physico-chemical attributes of the semi-permeable cytoplasmic membrane (Wood, 2011; Van den Berg et al., 2017; Bremer and Krämer, 2019). As a result, the bacterial cell faces dehydration at high environmental osmolarity, and concomitantly encounters an undesired increase in molecular crowding and a reduction of turgor to values unsuitable for efficient growth. Many microorganisms counteract the outflow of water through a sustained accumulation of compatible solutes (Brown, 1976). These organic osmolytes are compliant with the biochemistry and physiology of bacterial cells (Bolen and Baskakov, 2001; Ignatova and Gierasch, 2006; Stadmiller et al., 2017), and can thus be amassed to very high intracellular pools in a finely-tuned fashion in a response to the degree of osmotic stress imposed onto the cell. Accordingly, the accumulation of compatible solutes, either through synthesis or import, promotes cellular hydration and growth under osmotically unfavorable conditions (Kempf and Bremer, 1998; Roesser and Müller, 2001; Wood et al., 2001).

L-proline is a prominent member of the compatible solutes and is widely used by both plants and microorganisms as an osmostress protectant and chemical chaperone (Csonka, 1989; Cayley et al., 1992; Chattopadhyay et al., 2004; Ignatova and Gierasch, 2006; Fichman et al., 2014). The Gram-positive soil bacterium *Bacillus subtilis* is one of the microorganisms that uses the accumulation of L-proline to counteract high osmolarity and high salinity incurred water stress (Whatmore et al., 1990; Von Blohn et al., 1997; Brill et al., 2011a; Hoffmann et al., 2017). The genetic disruption of osmostress-adaptive L-proline biosynthesis causes osmotic sensitivity of *B. subtilis* (Brill et al., 2011a), thereby attesting to the physiological importance of L-proline accumulation for the cell’s ability to cope with osmotically challenging growth conditions (Hoffmann and Bremer, 2016). L-proline is the only compatible solute that *B. subtilis* can synthesize *de novo* (Whatmore et al., 1990; Brill et al., 2011a), as the synthesis of the osmostress protectant glycine betaine by this microorganism requires the prior import of the precursor molecule choline (Boch et al., 1994, 1996). Other members of the genus *Bacillus* can either rely exclusively on the synthesis of L-glutamate, or produce the compatible solutes ectoine/5-hydroxyectoine to counteract osmotic stress (Kuhlmann and Bremer, 2002; Bursy et al., 2007). Depending on the type of compatible solute synthesized by a given *Bacillus* species, different degrees of osmostress tolerance are attained. L-proline typically affords a substantial, yet intermediate, level of osmotic stress tolerance (Hoffmann and Bremer, 2016).

**Figure 1:**
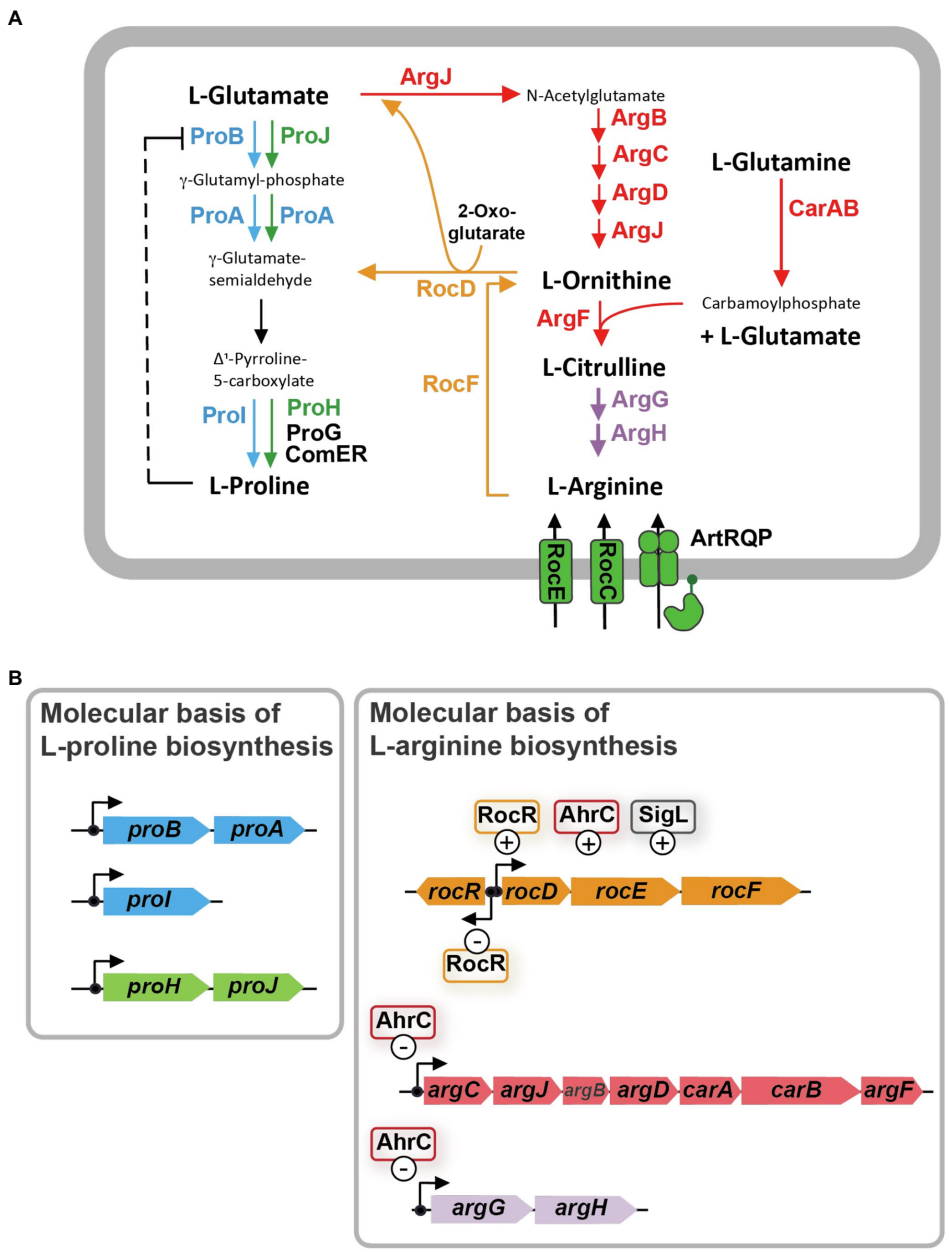
L-arginine biosynthesis and degradation pathways are metabolically interconnected with the L-proline biosynthetic route in *Bacillus subtilis*. **(A)** Schematic overview of the precursors, intermediates, and products of L-arginine synthesis and catabolism, and the anabolic and osmostress-responsive L-proline biosynthetic route. The ProB-ProA-ProI enzymes (blue) are used for anabolic L-proline production, while the ProJ-ProA-ProH (green) route is employed for the synthesis of L-proline as an osmostress protectant. γ-glutamate-semialdehyde spontaneously converts to Δ^1^-pyrroline-5-carboxylate which is the substrate for the Δ^1^-pyrroline-5-carboxylate reductase; four of these types of enzymes operate in *B. subtilis*. The precise physiological functions of the ProG and ComER enzymes are not entirely clear. **(B)** Molecular and regulatory overview of L-proline and L-arginine biosynthetic genes and those for L-arginine degradation. Positive and negative influence of known regulatory proteins on gene expression are highlighted. These data for this figure were compiled from the literature.

Synthesis of L-proline by bacteria typically proceeds from the central metabolite L-glutamate and is mediated by three enzymes: γ-glutamyl kinase (ProB), γ-glutamyl phosphate reductase (ProA), and Δ^1^-pyrroline-5-carboxylate reductase (ProC), with γ-glutamyl phosphate and γ-glutamate-semialdehyde/Δ^1^-pyrroline-5-carboxylate as the respective intermediates (Fichman et al., 2014). *Bacillus subtilis* adheres to this L-proline biosynthetic scheme (Belitsky et al., 2001). However, it possesses several L-proline biosynthetic isoenzymes (Figure 1A). Their activities and the transcription of their structural genes are differently regulated so that L-proline pools adequate for either anabolic or osmostress-protective purposes can be produced (Whatmore et al., 1990; Brill et al., 2011a,b; Forlani et al., 2017; Hoffmann et al., 2017). In order to synthesize the large quantities of L-proline under osmotic stress conditions, *B. subtilis* and related species need to re-route their metabolism to provide adequate cellular pools for the L-proline biosynthetic precursor L-glutamate (Kohlstedt et al., 2014; Godard et al., 2020). This central metabolite is successively drained as the environmental osmolarity increases and enhanced L-proline synthesis commences (Brill et al., 2011a).

Biosynthesis of L-proline is energetically costly (Akashi and Gojobori, 2002). Hence, *B. subtilis* uses both genetic and biochemical control mechanisms to tie anabolic L-proline production *via* the ProB-ProA-ProI route to the ongoing protein biosynthetic activities of the cell. At the genetic level, expression of the genes for the *proBA* operon encoding γ-glutamyl kinase (ProB) and γ-glutamyl phosphate reductase (ProA), and that of the gene (*proI*) encoding the Δ^1^-pyrroline-5-carboxylate reductase ProI (Figure 1B) are controlled by a T-box regulatory mechanism. This tRNA^Pro^-responsive riboswitch (Kreuzer and Henkin, 2018) allows only enhanced full-length transcription of the *proBA* operon and that of the *proI* gene when the L-proline pool is insufficient to adequately fuel protein biosynthesis (Brill et al., 2011b). At the biochemical level, feed-back inhibition of ProB enzyme activity by L-proline ensures that the flow of the precursor L-glutamate into the L-proline biosynthetic pathway (Figure 1A) is curbed when the L-proline pool is sufficiently high to promote growth (Fujita et al., 2003; Chen et al., 2006; Perez-Arellano et al., 2010). As a result of these combined genetic and biochemical regulatory mechanisms, the steady-state pool of L-proline is kept by non-osmotically stressed *B. subtilis* cells in a rather narrow range (about 10–20 mM; Whatmore et al., 1990; Brill et al., 2011a; Zaprasis et al., 2013a; Hoffmann et al., 2017).

In contrast, and depending on the severity of the environmentally imposed osmotic stress, *B. subtilis* amasses several hundred mM of L-proline to increase the osmotic potential of the cytoplasm in order to counteract water outflow (Whatmore and Reed, 1990; Brill et al., 2011a; Zaprasis et al., 2013a; Hoffmann et al., 2017). To provide these large quantities of L-proline, *B. subtilis* developed an osmostress-responsive L-proline biosynthetic pathway that is freed from the genetic and biochemical constraints imposed onto the anabolic route (Brill et al., 2011a; Hoffmann et al., 2017). The osmostress adaptive L-proline biosynthetic route (ProJ-ProA-ProH) consists of isoenzymes of the first (ProJ) and last (ProH) step of the anabolic L-proline biosynthetic route (ProB/ProI) but shares with it [for unknown reasons (Hoffmann et al., 2017)] the γ-glutamyl phosphate reductase (ProA; Figure 1A). The *proHJ* operon lacks a T-box, and instead, its transcription is strongly upregulated in response to high osmolarity or salinity (Brill et al., 2011a; Hoffmann et al., 2017). Furthermore, the feed-back control of the enzyme activity of ProJ is probably abolished, or at least strongly reduced, in comparison with the ProB isoenzyme (Fujita et al., 2003; Chen et al., 2006; Perez-Arellano et al., 2010). Despite the T-box control of *proBA*, enough ProA enzymes are produced in osmotically challenged cells to functionally match the amounts of the ProJ-ProH enzymes needed to produce large amounts of the osmostress protectant L-proline (Hoffmann et al., 2017). In this context, it is noteworthy that *Bacillus* species other than *B. subtilis* (e.g., *Bacillus licheniformis*, *Bacillus megaterium*) also possess two separate, yet biochemically complete, routes for either anabolic or osmostress protective L-proline production (Schroeter et al., 2013; Godard et al., 2020).

It is well known that the biosynthesis of L-proline and L-arginine are interconnected in many bacteria, including *B. subtilis* (Figure 1A; Belitsky, 2002; Fischer and Debarbouille, 2002; Csonka and Leisinger, 2007). As a result, suppressor mutations located in genes for L-arginine metabolism can bypass particular genetic blocks in L-proline biosynthesis (Itikawa et al., 1968; Kuo and Stocker, 1969; Berg and Rossi, 1974; Rossi et al., 1977). L-proline and L-arginine are both produced from the central metabolite L-glutamate, and γ-glutamate-semialdehyde/Δ^1^-pyrroline-5-carboxylate is an common intermediate in their respective biosynthetic routes (Baumberg and Harwood, 1979; Belitsky, 2002; Fischer and Debarbouille, 2002; Fichman et al., 2014; Figure 1A).

The γ-glutamate-semialdehyde/Δ^1^-pyrroline-5-carboxylate-dependent metabolic shunt between the L-arginine and L-proline biosynthetic routes operates very inefficiently in *B. subtilis* wild-type cells. This is evidenced by the fact that a *proA* mutant forms only tiny colonies on L-proline-free minimal medium agar plates with glucose as the carbon and ammonium as the nitrogen source (Zaprasis et al., 2013b). However, spontaneously occurring suppressor strains with increased growth performance can readily be isolated. In these suppressors, the genetic block in the ProA-catalyzed step is bypassed through a transcriptional up-regulation of the L-arginine catabolic *rocDEF* operon (Figure 1A; Zaprasis et al., 2013b). This allows the *rocD*-encoded ornithine aminotransferase (Figure 1B) to convert increased amounts of L-ornithine, an intermediate in L-arginine biosynthesis, to produce increased amounts of γ-glutamate-semialdehyde/Δ^1^-pyrroline-5-carboxylate, metabolites that are also the product(s) of the ProA enzyme (Figure 1A; Belitsky et al., 2001; Fichman et al., 2014). Hence, ProA enzyme activity can be bypassed as Δ^1^-pyrroline-5-carboxylate can be converted into L-proline by Δ^1^-pyrroline-5-carboxylate reductase, a type of enzyme present in *B. subtilis* in four different forms (ProI, ProH, ProG, ComER; Figure 1A; Belitsky et al., 2001; Brill et al., 2011a; Forlani et al., 2017; Hoffmann et al., 2017). Accordingly, only a *proA rocD* double-mutant of *B. subtilis* exhibits a tight L-proline auxotrophic growth phenotype (Zaprasis et al., 2013b).

While the previously reported suppressor strains of a *proA* defect in *B. subtilis* allow the production of L-proline pools sufficiently large to restore growth in a L-proline free minimal medium, none of them was able to attain osmostress protective cellular levels of L-proline (Zaprasis et al., 2013b). Building on the idea that microorganisms can almost always find a way to circumvent metabolic constraints through mutational changes (Barrick and Lenski, 2013), we have now investigated if *proA* bypass suppressors can be found that would restore osmostress tolerance to *B. subtilis*. Indeed, we found such suppressor strains, and in each of these mutants the same physiological process, L-arginine biosynthesis, was targeted, albeit as the consequence of different genetic events. Our analysis of the metabolome of some of these osmostress tolerant suppressors suggests that not only L-proline but also L-citrulline might function as a compatible solute for *B. subtilis* under special circumstances.

## Materials and Methods

### Chemicals, Growth Media, and Culture Conditions

All antibiotics used in this study and the chromogenic substrate (para-nitrophenyl-α-glycopyranoside; α-PNPG) for activity assays of the TreA enzyme, a phospho-α-(1,1)-glucosidase, were obtained from Sigma-Aldrich (Steinheim, Germany). *Bacillus subtilis* strains were maintained at room temperature on LB-agar plates. Liquid cultures of *B. subtilis* strains were grown in shake flasks (20 ml culture volume in 100-ml Erlenmeyer flasks) set in a rotating water bath (220 rpm at 37° C) until they reached an OD_578_ indicated in the individual experiments. Throughout our study, we used Spizizen’s minimal medium (SMM) with glucose (0.5% w/v) as the carbon source, ammonium as the nitrogen source, and a solution of trace elements (Harwood and Archibald, 1990). Because the *B. subtilis* wild-type strain JH642 (Smith et al., 2014) and its derivatives (Table 1; Supplementary Table S1) are auxotrophic for L-phenylalanine and L-tryptophan, these two amino acids were added to the growth medium (L-Phe at 18 mg L^−1^ and L-Trp at 20 mg L^−1^). When high-salinity growth medium was used, pre-cultures were grown in SMM without additional NaCl to early-exponential growth phase (OD_578_ of about 1.5) and were then used to inoculate main cultures, to an OD_578_ of about 0.1, containing increased NaCl concentrations as indicated in the individual experiments.

**Table 1:** Strains used in this study.

Strain^a^	Genotype^b^	Suppressor name	Osmotic stress resistance^c^	References
JH642	*pheA*1 *trpC*2		+	BGSC 1A96^d^
JSB8	JH642 Δ(*proHJ*::*tet*)1		−	Brill et al., 2011a
GWB120	JH642 Δ(*proBA*::*cat*)2 (P*_rocD_*-P1)	Pro^+^-1	−	Zaprasis et al., 2013b
DRB16	JH642 Δ(*proBA*::*cat*)2 (P*_rocD_*-P1) P*_argC_*-O4	Pro^+^-20	+	This work
DRB17	JH642 Δ(*proBA*::*cat*)2 (P*_rocD_*-P1) *ahrC*21	Pro^+^-21	+	This work
GWB128	JH642 Δ(*proBA*::*cat*)2 *rocR-*9	Pro^+^-9	−	Zaprasis et al., 2013b
DRB28	JH642 Δ(*proBA*::*cat*)2 *rocR-*9 P*_argC_*-O4	Pro^+^-32	+	This work
DRB30	JH642 Δ(*proBA*::*cat*)2 *rocR-*9 *ahrC*34	Pro^+^-34	+	This work
DRB4	JH642 Δ(*proA*::*ery*)		−	This work
DRB40	JH642 Δ(*proA*::*ery*) P*_argC_*-O7	Pro^+^-43	−	This work
DRB42	JH642 Δ(*proA*::*ery*) *ahrC-*45	Pro^+^-45	−	This work

a All strains are derivatives of the *B. subtilis* strain JH642. Its genome sequence has been reported (Smith et al., 2014).

b The P*_rocD_*-P1 allele activates a cryptic SigA-type promoter in front of the *rocDEF* operon. The *rocR-*9 allele (L250/H) causes a single amino acid substitution in the RocR regulatory protein making it partially inducer-independent (Zaprasis et al., 2013b). The P*_argC_*-O4 and P*_arg_C*-O7 alleles are point mutations in the main AhrC operator sequence of the *argCJBD-carAB-argF* operon. *ahrC-*34 causes a single amino acid substitution in the AhrC regulatory protein, while the *ahrC-*45 allele introduces a frame-shift mutation into the *ahrC* gene. Loss of the *proB*-encoded γ-glutamyl kinase enzyme is compensated for by the corresponding activity of the ProJ enzyme, while only a single *proA*-type gene is present; hence there is only a single γ-glutamyl phosphate reductase active in *B. subtilis* (Belitsky et al., 2001; Brill et al., 2011a).

c The symbol “+” indicates that the particular strain can grow on SMM plates containing 0.8 M NaCl, while the symbol “−” indicates that no efficient growth occurs (see Figure 3).

d BGSC: Bacillus Genetic Stock Center, Ohio, United States.

To verify integration of P*argC-treA* (*cat^R^*) reporter fusion constructs *via* a double recombination event into the chromosomal *amyE* gene as a single copy (Harwood and Archibald, 1990), corresponding *B. subtilis* strains were grown on LB-agar plates containing 1% starch. The plates were then flooded with Gram’s iodine stain for 1 min and scored for zones of starch degradation around colonies to identify those transformants that were no longer able to degrade starch (the AmyE phenotype; Harwood and Archibald, 1990). To select chloramphenicol resistant (*cat^R^*) *B. subtilis* strains after transformation with linearized plasmid DNA encoding various P*argC-treA* transcriptional reporter constructs, LB agar plates containing 5 μg of this antibiotic were used. P*argC-treA* reporter fusion derivatives of plasmid pPINK1 (Supplementary Table S2) were selected in the *Escherichia coli* strain TOP10 (Invitrogen, Darmstadt, Germany), by plating competent cells on LB agar plates containing 100 μg ml^−1^ of ampicillin.

### Recombinant DNA Procedures, Construction of Plasmids and of *Bacillus subtilis* Strains

**Figure 2:**
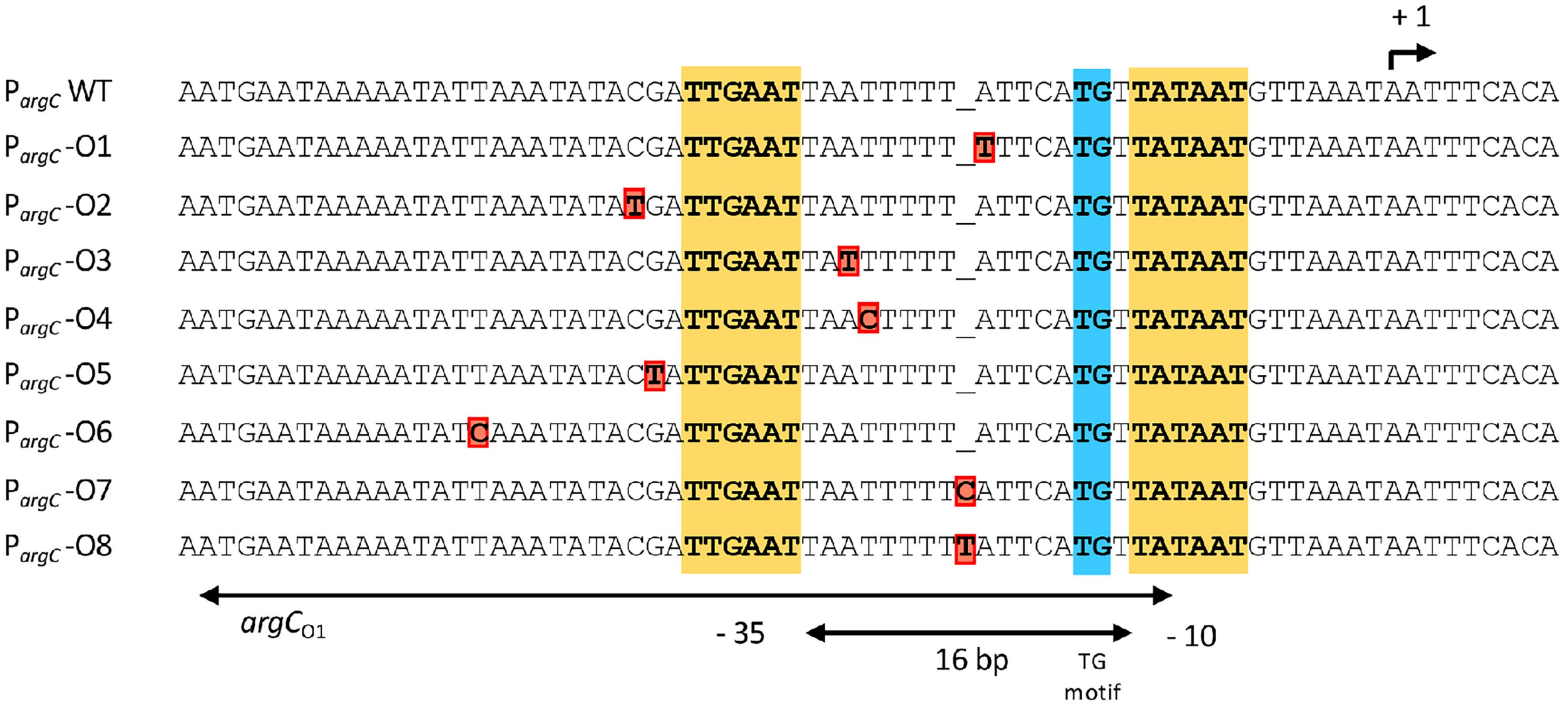
DNA sequence of the *argC* promoter region and that of the overlapping main operator for the AhrC regulatory protein. AhrC operator mutations derived from the salt-stress resistance suppressor screen are indicated in red. The −35 and of the extended −10 regions (with a TG motif) of the *argC* promoter are highlighted. The *argC* promoter possesses a sub-optimal spacing of 16 bp in comparison with typical SigA-type promoters of *B. subtilis* (Helmann, 1995). The transcriptional start site for the *argCJBD-carAB-argF* operon is indicated by a bent arrow. The AhrC operator(s) and its interactions with the AhrC regulatory protein, which is a homohexamer, have been defined through molecular and structural analysis (Garnett et al., 2007a, 2008). Three ArgC operators are present to transcriptionally control *argCJBD-carAB-argF* expression. One of these is present in the coding region of the *argC* gene, while the two other operators are positioned such that one of them overlaps the −10 and −35 regions of the SigA-type promoter. The other operator is positioned further upstream, with a 11 bp DNA sequence separating them. Binding of AhrC to the operators overlapping with and juxta positioned to the *argC* promoter leads to the bending of the DNA (Garnett et al., 2008). The entire regulatory region has been termed *argC*_O1_ while the ArgC operator present in *argC* is referred to as *argC*_02_ (Czaplewski et al., 1992).

All recombinant DNA work used established procedures. To construct transcriptional fusions between the *argC* regulatory region to the promoter-less *treA* reporter gene (Schöck et al., 1996), a 427 bp DNA fragment of the wild-type sequence and of six AhrC-operator mutants (Figure 2) was amplified by PCR using the DNA primers DS27_5′-ATTGGGCCCGAGTGGATTGATGATGATGA-3′ and DS28_5′-ATTGGATCCTCGTATTCATATCAATCGGGC-3′. The amplified fragment carrying the *argC* promoter and its AhrC responsive regulatory sequences was inserted in front of the promoter-less *treA*^+^ gene carried by the low-copy number plasmid pPINK1 that had been cut with the restriction enzymes BamHI and SmaI. The resulting P*argC-treA* reporter plasmids are listed in Supplementary Table S2. The DNA of each of these plasmids was linearized by cutting with the restriction enzymes XhoI and PstI and was then used to transform the *B. subtilis* strain GNB37 [(*treA::ery*)2 *argC*^+^)] (Nau-Wagner et al., 2012). Each of the P*argC-treA* constructs is followed by a chloramphenicol resistance gene (*cat^R^*), and the entire cassette is flanked by 5′ and 3′ segments of the *amyE* amylase gene. This allows the selection of recombinant strains *via* their chloramphenicol resistance and the identification of chromosomal *amyE*::P*argC-treA-cat^R^::amyE* insertions by scoring the AmyE^−^ phenotype on starch plates. To combine the various P*argC-treA* reporter fusions with an *ahrC* gene disruption mutation, we used DNA of the linearized reporter plasmids to transform strain DRB47 (Δ*ahrC::ery treA::kan*) by selecting chloramphenicol-resistant colonies and scoring their EmyE^−^phenotype on starch plates. The resulting strains are listed in Supplementary Table S1. To combine the wild-type P*argC-treA* transcriptional reporter fusion with various *ahrC* suppressor alleles (Table 2), we used DNA of linearized plasmid pDST40 (*argC-treA*) to transform corresponding suppressor strains, again selecting chloramphenicol-resistant colonies. The resulting strains are listed in Supplementary Table S1.

**Table 2:** Suppressor mutations targeting the *ahrC* regulatory gene.

GWB120 background [∆*proBA* P*_rocD_*-P1]		GWB128 background [∆*proBA rocR-*9]		DRB4 background [∆*proA*]	
Mutation in *ahrC*		Mutation in *ahrC*^b^		Mutation in *ahrC*^b^	
Pro^+^-21^a^	Frame shift in codon 26	Pro^+^-34^a^	T40K	Pro^+^-45	Frame shift in codon 143
		Pro^+^-35	Q38H	Pro^+^-46	A103D
		Pro^+^-36	G101D		
		Pro^+^-42	Frame shift in codon 120		

a Suppressors used for detailed further studies.

b Pro^+^-21 codon GTC^26^ to ∆TC; Pro^+^-34 codon ACG^40^ to AAG; GCC^103^ to GAC; Pro^+^-35 codon CAG^38^ to CAT; Pro^+^-36 GGC^101^ to GAC; Pro^+^-42 GGG^120^ to GG∆; Pro^+^-45 AAC^143^ to AA(A)C; Pro^+^-46 GCC^103^ to GAC.

### TreA Reporter Enzyme Activity Assays

Promoter activities of P*argC-treA* transcriptional reporter fusions inserted into the *B. subtilis* genome at the *amyE* gene were measured as described before (Schöck et al., 1996; Brill et al., 2011a; Zaprasis et al., 2013b). *treA* encodes a salt-tolerant phospho-α-(1,1)-glucosidase whose enzyme activity can be quantitated using the chromogenic synthetic substrate α-PNPG (Schöck et al., 1996). *Bacillus subtilis* strains carrying P*argC-treA* transcriptional fusions all harbored a gene disruption of the native *treA* gene (Supplementary Table S1), so that the measured TreA enzyme reporter activity reflects exclusively that of the reporter gene construct. To measure the promoter activities of the various P*argC-treA* transcriptional fusions, strains were grown in SMM at 37° C (20 ml culture volume in 100-ml Erlenmeyer flasks) in the absence or presence of 20 mM L-arginine (Gardan et al., 1997) until they reached an OD_578_ of 1.5. 1.8-ml samples were withdrawn from the culture and assayed for TreA enzyme activity as described (Brill et al., 2011a; Zaprasis et al., 2013b).

### Genome Re-sequencing and Targeted Analysis of *Bacillus subtilis* Strain JH642 Mutant Derivatives

**Figure 3:**
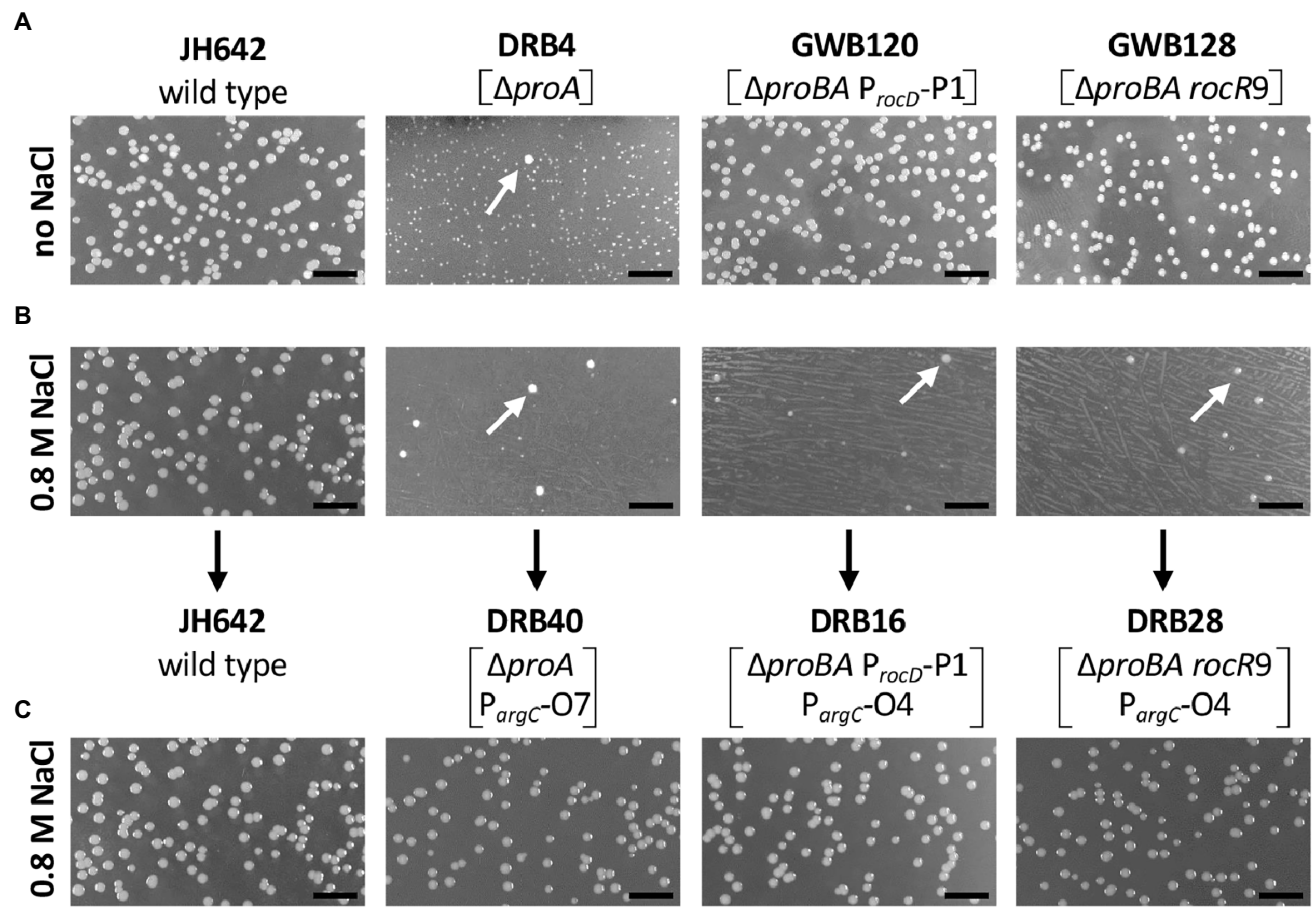
Selection for osmostress resistant suppressor mutants bypassing a defect in the *proA*-encoded γ-glutamyl phosphate reductase. Equal dilutions of the indicated strains were plated onto either **(A)** SMM agar plates lacking L-proline or **(B)** SMM agar plates lacking L-proline but that contained 0.8 M NaCl. The plates were incubated for 72 h at 37° C. **(C)** Spontaneously arising faster growing suppressor colonies (indicated by the white arrows) were picked from the high-salinity agar plates, purified by streaking single colonies on the same medium, and dilution of liquid cultures were replated onto the high-salinity L-proline-free agar plates. The length bar corresponds to 5 mm. *Bacillus subtilis* JH642 is the wild-type strain (Smith et al., 2014) from which the first generation suppressor strains DRB4, GWB120 and GWB128 were derived (Zaprasis et al., 2013b). Strain DRB4 and its derivatives contain a deletion of the *proA* gene but *proB* is intact. In strains GWB120 and GWB128, the entire *proBA* operon is deleted but ProB enzyme activity is provided in these strains *via* the amino acid sequence related ProJ L-glutamate kinase; hence these strains are not L-proline auxotrophs (Belitsky et al., 2001; Brill et al., 2011a; Zaprasis et al., 2013b). Strains GWB120 and GWB128, carry mutations either in the regulatory region of the *rocDEF* operon, or in the gene for the RocR activator protein; both mutations increase *rocDEF* expression (Zaprasis et al., 2013b).

To identify the suppressor mutations in the evolved *B. subtilis proA* suppressor strains (Figures 3B,C), genomic DNAs were subjected to DNA sequencing, which was kindly performed by the Göttingen Genomics Laboratory (Göttingen, Germany) on Illumina instruments. The reads were mapped onto the reference genome sequence of the *B. subtilis* strain JH642 (GenBank accession number CM000489.1; Smith et al., 2014) as previously described (Widderich et al., 2016) using the Geneious software package (Kearse et al., 2012). Single nucleotide polymorphisms were considered as significant when the total coverage depth exceeded 25 reads with a frequency variance of >90%. The genome sequence of the following salt-tolerant suppressor strains was determined: DRB16, DRB17, DRB20, DRB28, and DRB40 (Table 1; Supplementary Table S1).

In addition to suppressor mutants whose entire genome was re-sequenced, we obtained the DNA sequence of the *ahrC* gene and of the *argC* regulatory region of the *argCJBD-carAB-argF* operon in 22 suppressor stains by amplifying the corresponding DNA segment *via* PCR (*ahrC*: 715 bp; *argC*: 598 bp) and subsequent DNA sequence analysis. The DNA sequence of the DNA primers used to amplify the *ahrC* gene were: DS23_5’-TGCGCGTTGTAGAAGAAGCA-3′ and DS24_5’-GCCCGCGTTCAAAAGAAACC-3′. Those used for the amplification of the *argC* regulatory region were DS25_5’-CCATTATGCTCGGGGGCTTT-3′ and DS26_5’-AACCGTAATTCCCGCGTCTG-3′. DNA sequencing was carried out by Eurofins MWG Operon (Ebersberg, Germany).

### Quantitative Metabolomics

To detect and quantify metabolites of the L-proline and L-arginine biosynthetic pathways, we used targeted metabolomics. For these experiments, two independently prepared cultures of the *B. subtilis* strain JH642 and its mutant derivatives were grown in SMM or SMM containing 1.2 M NaCl. Cells were cultured in shake flasks (20 ml culture volume in 100-ml Erlenmyer flasks) to early-exponential growth phase (OD_578_ of about 1.5). 1 ml culture aliquots were withdrawn from the cultures and the cells were rapidly vacuum-filtered onto a 0.45 μm pore size filter (HVLP02500, Merck Millipore). The filters were immediately transferred into a acetonitrile/methanol/water (40:40:20) extraction solution at −20°C. The filters were incubated in the extraction solution for 30 min. Subsequently, the metabolite extracts were centrifuged for 15 min at 13,000 rpm at −9° C and the supernatant was stored at −80° C until analysis.

Metabolite extracts were mixed with a ^13^C-labeled internal standard in a 1:1 ratio. LC-MS/MS analysis was performed with an Agilent 6495 triple quadrupole mass spectrometer (Agilent Technologies) as described previously (Guder et al., 2017). An Agilent 1290 Infinity II UHPLC system (Agilent Technologies) was used for liquid chromatography. The temperature of the column oven was 30°C, and the injection volume was 3 μl. LC solvents in channel A were either water with 10 mM ammonium formate and 0.1% formic acid (v/v; for acidic conditions), or water with 10 mM ammonium carbonate and 0.2% ammonium hydroxide (for basic conditions). LC solvents in channel B were either acetonitrile with 0.1% formic acid (v/v; for acidic conditions) or acetonitrile without additive (for basic conditions). LC columns were an Acquity BEH Amide (30 × 2.1 mm, 1.7 μm) for acidic conditions, and an iHILIC-Fusion(P; 50 × 2.1 mm, 5 μm) for basic conditions. The gradient for basic and acidic conditions was: 0 min 90% B; 1.3 min 40% B; 1.5 min 40% B; 1.7 min 90% B; 2 min 90% B. The ratio of ^12^C and ^13^C peak heights was used to quantify metabolites and absolute concentrations were determined by calibrating the ^13^C standard with authentic standards. Intracellular concentrations of metabolites were calculated using an intracellular volume of 0.65 μl per 1 ml *B. subtilis* culture grown to an OD_578nm_ = 1. The *B. subtilis* cell volume was estimated from previously reported values for the internal and total water spaces that were determined by measuring the distribution of membrane-permeable ^3^H_2_O and membrane-impermeable inulin-[^14^C]carboxylic acid (Bakker and Mangerich, 1981; Holtmann et al., 2003; Hoffmann et al., 2013). The cellular extracts of two biological replicates were assessed and each of them was measured in at least two technical parallels.

### Statistical Assessment

**Figure 4:**
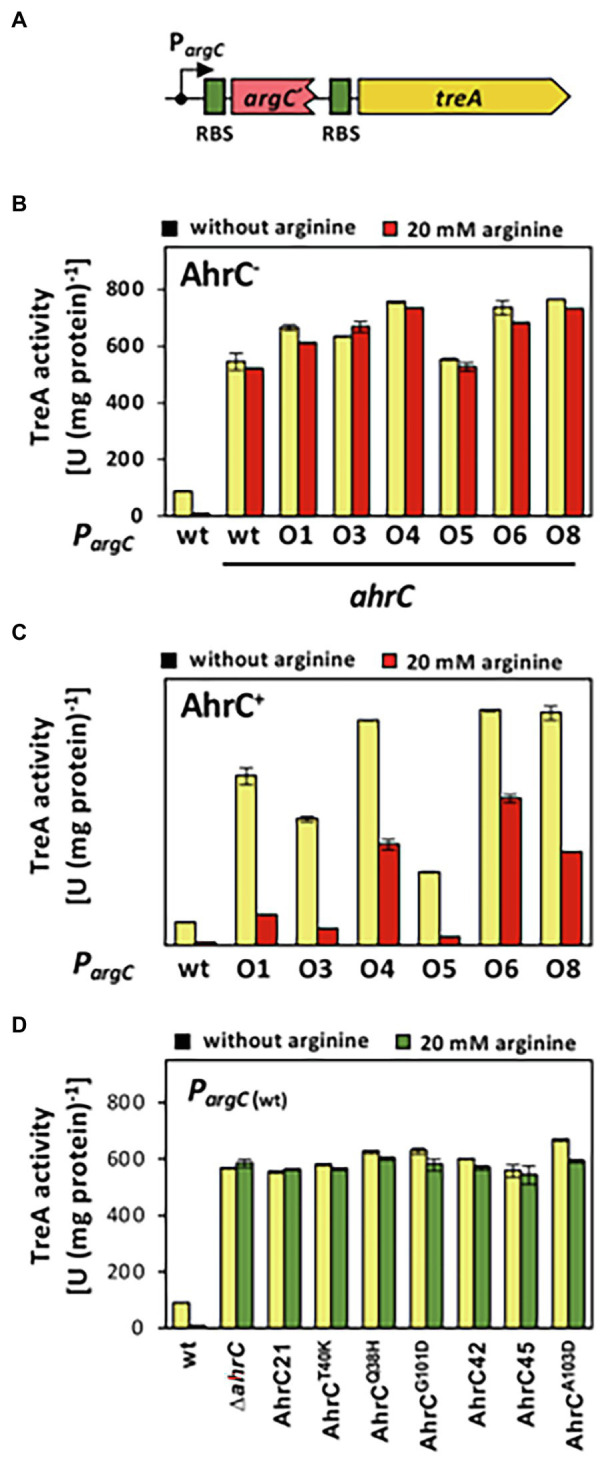
Reporter gene assays assessing the influence of *argC* operator or *ahrC* regulator mutations on *argC* promoter activity. **(A)** Scheme of a P*argC-treA* transcriptional reporter fusion. The *treA* gene encodes a salt-tolerant a phospho-α-(1,1)-glucosidase (Schöck et al., 1996). In the reporter fusion construct, *treA* lacks its own promoter and its transcription is thus dependent on the promoter activity mediating the expression of *the argCJBD-carAB-argF* operon. *treA* possesses its own ribosome binding site (RBS; green box). (B, C) The P*argC-treA* transcriptional fusion was stably integrated *via* a double recombination event as a single copy into the chromosomal *amyE* gene of an *ahrC* mutant **(B)**, or into the chromosome of the *ahrC*^+^ **FIGURE 4 | ***B. subtilis* wild-type strain JH642 **(C)**. Mutant derivatives of the P*argC-treA* reporter fusion strains carrying various mutations in the AhrC operator (O1 to O8) in the *argC-treA* construct were similarly constructed. **(D)** The wild-type P*argC-treA* transcriptional fusion was inserted either into the chromosome of the wild-type strain JH642, or into derivatives of this strain carrying various *ahrC* mutant alleles. For details on the types of *argC* operator mutants see Figure 2 and for the description the *ahrC* mutant alleles see Table 3. All strains carry a disruption of the chromosomal *treA* gene so that the measured TreA enzyme activities reflect solely that encoded by the P*argC-treA* reporter fusion. The shown data represent experiments from two biological replicates and each sample was assayed twice. SDs for TreA reporter enzyme activity are indicated by bars and the statistical significands of the reported values are listed in Supplementary Table S3.

**Figure 5:**
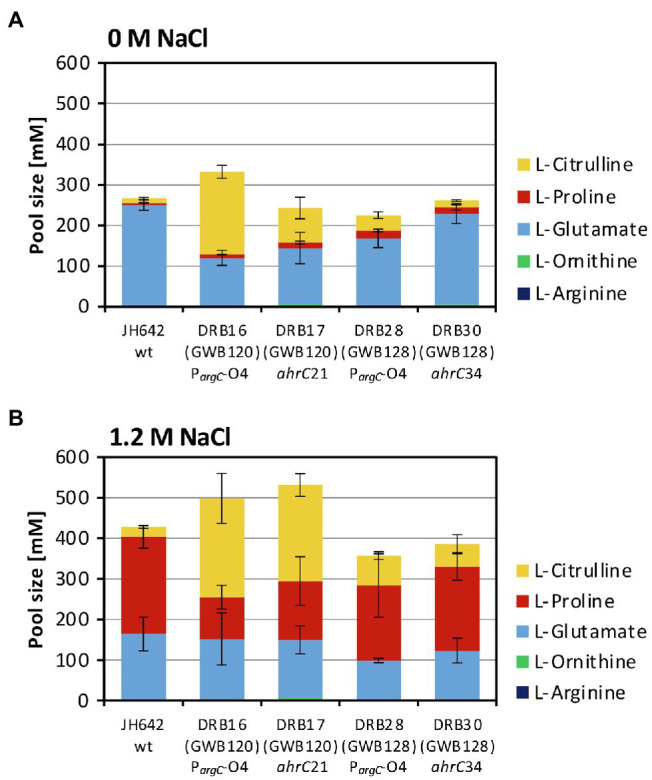
Targeted metabolome analysis of the *B. subtilis* wild-type strain and selected osmostress tolerant suppressor derivatives. Metabolites from the *B. subtilis* wild-type (WT) strain JH642 and of four osmostress tolerant suppressor derivatives were analyzed by focusing on L-proline and on key metabolites of the L-arginine synthesis and degradation intermediates. The corresponding metabolites were assessed and quantitated form cells that had been grown in SMM to early exponential growth phase (OD_578_ of about 1.5) containing either no **(A)** additional NaCl or **(B)** 1.2 M NaCl. The values given are averages and SDs of two biological replicates and each of the cellular extracts was measured in at least two technical parallels’. The precise values of the measured metabolites are documented in Supplementary Table S4. SDs for the measurements of the indicated metabolites are indicated by bars and the statistical significands of the reported values are listed in Supplementary Table S5.

Statistical assessment of values obtained for assays of the TreA reporter enzyme (Figures 4B–D) or individual compounds detected and quantitated in the course of targeted metabolomics (Figure 5; Supplementary Table S4), was carried out with the “unpaired t test” analysis tool as implemented in the Prism 9 software suite (GraphPad Software, San Diego, CA, United States; see Supplementary Tables S3, S5).^1^

## Results

### Selection of Osmostress Resistant Mutants Bypassing the ProA L-Proline Biosynthetic Bottleneck

Faster growing Pro^+^ suppressor strains arising spontaneously in a *proA* mutant fall genetically into two classes (Zaprasis et al., 2013b). In one class [represented by strain GWB120 (Δ*proBA* P*_rocD_*-P1); Figure 3A], point mutations activate a cryptic SigA-type promoter present in front of the *rocDEF* operon (Zaprasis et al., 2013b), a gene cluster whose transcription is otherwise dependent on the alternative Sig-54-type sigma factor SigL and the NtrC/NifA-type activator protein RocR (Debarbouille et al., 1991; Calogero et al., 1994; Gardan et al., 1995, 1997). In the second class of suppressor mutants (represented by strain GWB128 (Δ*proBA rocR*-9; Figure 3A), single amino acid substitutions in RocR result in partial effector-independent variants of this activator protein (Zaprasis et al., 2013b). Hence, enhanced *rocDEF* expression occurs even when the RocR effector molecules L-arginine, L-ornithine, or L-citrulline are not added to the growth media (Calogero et al., 1994; Gardan et al., 1995, 1997; Ali et al., 2003).

While the above described suppressor mutants allow wild-type level growth in an L-proline-free minimal medium (SMM; Figure 3A), none of them was able to produce L-proline pools sufficiently large to confer osmostress resistance (Zaprasis et al., 2013b). This is seen when the first generation of the suppressor strains are plated on SMM agar plates containing 0.8 M NaCl, conditions under which the wild-type *B. subtilis* strain JH642 can readily grow, while the suppressor strains cannot (Figure 3B). However, we observed that faster growing colonies spontaneously arose from the background lawn of strains DRB4 (Δ*proA*), GWB120 (Δ*proBA* P*_rocD_*-P1) and GWB128 (Δ*proBA rocR*-9; Table 1) when these strains were plated on a L-proline-free minimal medium with increased salinity (0.8 M NaCl; Figure 3B). Isolation and re-plating of the suppressor strains on high salinity SMM agar plates containing 0.8 M NaCl yielded colonies with a growth behavior and visual appearance resembling that of the *B. subtilis* wild-type strain JH642 (Figure 3C). Collectively, these observations indicated that the second generation of suppressor strains had gained the ability to withstand high salinity-incurred cellular stress, despite the fact that its natural biosynthetic route (ProJ-ProA-ProH; Brill et al., 2011a) for the osmostress protectant L-proline, the only compatible solute that *B. subtilis* can synthesize *de novo* (Whatmore et al., 1990; Brill et al., 2011a), was not intact due to the disruption of the *proA* gene (Figure 1A).

### Genome Re-sequencing and Targeted DNA-Sequence Analysis Reveals the Molecular Determinants for Increased Salt Tolerance of the Suppressor Strains

We chose four independently isolated suppressor strains with increased salt tolerance that were derived from strain DRB4 (Δ*proA*), 12 suppressor colonies derived from strain GWB120 (Δ*proBA* P*_rocD_*-P1) and 11 suppressor strains derived from strain GWB128 (Δ*proBA rocR*-9) for further analysis. The genome sequence of the *B. subtilis* laboratory strain JH642, a derivative of strain 168, is known (Smith et al., 2014). We therefore resorted to whole genome re-sequencing of the JH642-derived suppressor strains to reveal possible leads for the type(s) of mutations giving rise to increased salt stress resistance. To this end, we sequenced the genomes of five randomly picked strains from our collection of 27 suppressor isolates. Four strains carried single base-pair substitutions in the high-affinity operator sequence for the AhrC repressor protein overlapping the −10 and −35 promoter sequence of the *argCJBD-carAB-argF* arginine biosynthetic operon of *B. subtilis* (Smith et al., 1989; Czaplewski et al., 1992; Miller et al., 1997; Garnett et al., 2008). One strain carried a mutation in the *ahrC* regulatory gene (Dennis et al., 2002; Garnett et al., 2007a,b, 2008; Tables 2, 3). Mutations targeting the AhrC operator sequence (P*arg*-O1, P*arg*-O4, P*arg*-O7; Figure 2) were found among the suppressors derived from each of the three *B. subtilis* strains used for the original genetic selection scheme. The same point mutation (*Parg*-O4; Figure 2) was even present in suppressors derived either from strain GWB120 or from strain GWB128 (Table 3). As expected, the chromosome of the five suppressor strains chosen for genome re-sequencing also contained the mutations that were originally present in the parental strains. No other mutations outside of the AhrC operator or of the *ahrC* gene were found.

**Table 3:** Suppressor mutations targeting the AhrC operator of the *argCJBD-carAB-argF* L-ornithine/L-citrulline/L-arginine biosynthetic operon.

GWB120 background [∆*proBA* P*_rocD_*-P1]		GWB128 background [∆*proBA rocR-9*]		DRB4 background [∆*proA*]	
**Mutation in P**_***argC***_^a^		**Mutation in P**_***argC***_^a^		**Mutation in P**_***argC***_^a^	
Pro^+^-20	P_argC_-O4	Pro^+^-32	P_argC_-O4	Pro^+^-43	P_argC_-O7
Pro^+^-22	P_argC_-O1	Pro^+^-33	P_argC_-O5	Pro^+^-44	P_argC_-O8
Pro^+^-23	P_argC_-O2	Pro^+^-37	P_argC_-O6		
Pro^+^-24	P_argC_-O4	Pro^+^-38	P_argC_-O6		
Pro^+^-25	P_argC_-O4	Pro^+^-39	P_argC_-O5		
Pro^+^-26	P_argC_-O3	Pro^+^-40	P_argC_-O2		
Pro^+^-27	P_argC_-O3	Pro^+^-41	P_argC_-O2		
Pro^+^-28	P_argC_-O3				
Pro^+^-29	P_argC_-O3				
Pro^+^-30	P_argC_-O8				
Pro^+^-31	P_argC_-O3				

a Each of these suppressor alleles target the AhrC operator positioned in front of the *argCJBD-carAB-argF* operon (see Figure 2 for the types of mutations that have occurred).

While the number of suppressor strains subjected to whole genome re-sequencing is restricted, our data provided a consistent picture about the molecular underpinnings for the salt-stress-resistant phenotype of the suppressor strains. In each of them, the same genetic process was targeted; namely the genetic control of the biosynthetic route for L-arginine and for its main intermediates, the non-proteinogenic amino acids L-ornithine and L-citrulline (Figure 1A). To corroborate this conclusion further, we amplified by PCR the regulatory region of the *argCJBD-carAB-argF* operon and of the *ahrC* regulatory gene from the remaining 22 strains of our suppressor collection. In each case, we found a point mutation in either of these DNA regions. Seventeen strains carried mutations in the AhrC operator sequence positioned in front of the *argCJBD-carAB-argF* gene cluster (Miller et al., 1997; Garnett et al., 2008; Table 3). This increased the total number of different mutations in the AhrC operator sequence up to eight (Figure 2). Additional six mutations occurred in the *ahrC* gene. Among the seven *ahrC* variants that we recovered in our genetic suppressor screen, four single amino acid substitution mutations were found, and three mutations led to truncated AhrC proteins caused by frame-shift mutations (Table 2).

**Figure 6:**
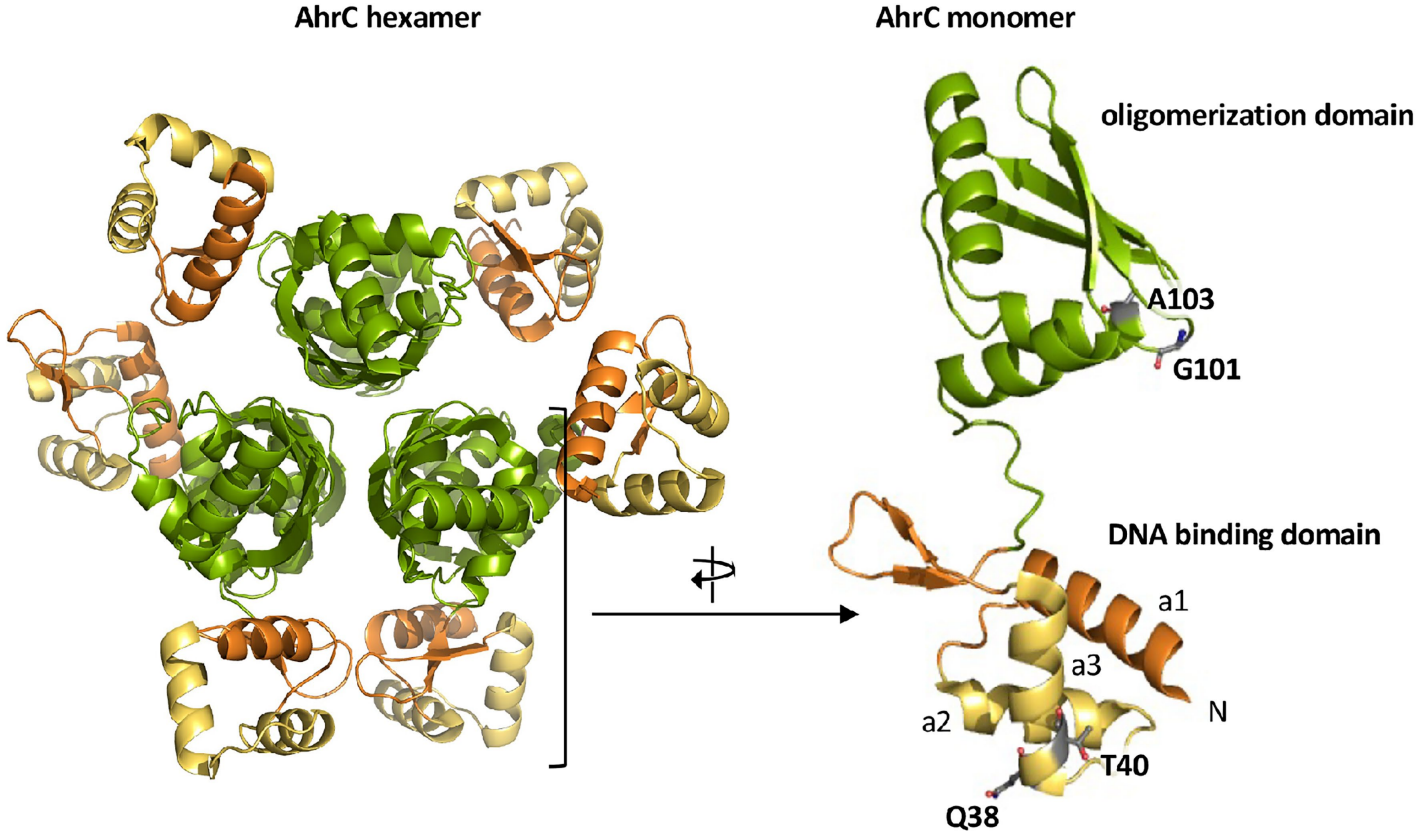
Crystal structure of the AhrC regulatory protein. The *B. subtilis* AhrC protein is a homo-hexamer and its monomer possesses two functionally distinct domains that are connected by a flexible linker region (Dennis et al., 2002). The N-terminal domain is involved in DNA-binding and contains a winged helix-turn-helix DNA-binding motif (yellow); the C-terminal part represents the effector (L-arginine) and multimerization domain (green; Garnett et al., 2007a,b). The representation of the AhrC hexamer and monomer was rendered using PyMol (Delano, 2002) and by using structural information deposited in the RCSB Protein Data Bank (pdb code: 1F9N). The positions of four single amino acid substitutions in AhrC leading to an osmostress-tolerant growth phenotype (Table 3) are indicated.

The *B. subtilis* AhrC regulatory protein serves as a repressor for the L-arginine biosynthetic gene clusters *argCJBD-carAB-argF* and *argGH* (Czaplewski et al., 1992; Figures 1A,B). However, it also functions as a transcriptional activator for the L-arginine catabolic operons *rocABC* and *rocDEF* (Gardan et al., 1995; Klingel et al., 1995; Figure 1B). The *B. subtilis* AhrC protein comprises 149 amino acids and the crystal structure of this hexameric protein (Figure 6) is known (Dennis et al., 2002). The monomer adopts a two-domain structural organization comprising an N-terminal DNA-reading head that contains a winged helix-turn-helix DNA binding motif, while the C-terminal domain mediates oligomerization into a hexamer and binding of the corepressor molecule L-arginine (Figure 6; Garnett et al., 2007a,b).

Four of the *ahrC* suppressor alleles that we recovered encode AhrC variants containing single amino acid substitutions (Table 2). Two of these (Q38/A and T40/K) are present in the winged helix-turn-helix-DNA binding motif of AhrC, while the second pair (G101/D and A103/D) are located in the oligomerization and L-arginine effector-binding domain (Figure 6; Czaplewski et al., 1992; Garnett et al., 2007b, 2008).

### Transcriptional Analysis of the *argC* Operator Mutations Conferring Increased Salt Tolerance

So far, we have assumed that the *argC* operator mutations (Figure 2) will lead to enhanced expression of the *argCJBD-carAB-argF* arginine biosynthetic operon (Smith et al., 1989; Czaplewski et al., 1992; Garnett et al., 2008). To show this directly, we constructed a set of transcriptional reporter fusions by linking the regulatory region of the *argCJBD-carAB-argF* gene cluster to a promoter-less *treA* reporter gene, which encodes a salt-tolerant phospho-α-(1,1)-glucosidase (Schöck et al., 1996). We stably inserted these P*argC-treA* transcriptional reporter gene constructs (Figure 4A) in single copy into the chromosomal non-essential *amyE* gene of *B. subtilis* through a double-homologous recombination event. We then measured TreA reporter enzyme activity in cells of these strains grown in SMM with glucose as the carbon source either in the absence or the presence of 20 mM L-arginine (Gardan et al., 1997). L-arginine serves as an AhrC-dependent co-repressor for *argCJBD-carAB-argF* transcription (Garnett et al., 2007b). The presence of increased L-arginine pools signals the *B. subtilis* cell that only low transcriptional levels of the corresponding biosynthetic operon are required to sustain adequate protein biosynthesis and hence growth (Czaplewski et al., 1992; Garnett et al., 2008). Several L-arginine import systems are present in *B. subtilis* (Figure 1A) so that changes in the transcription of the *argCJBD-carAB-argF* operon can be triggered by adding L-arginine to the growth medium.

When the wild-type P*argC-treA* transcriptional reporter construct was inserted into a *B. subtilis* strain proficient in AhrC, the reporter fusion was expressed at moderate levels, and the presence of L-arginine in the growth medium largely repressed promoter activity (Figure 4A). Conversely, transcription of the same fusion was about 50-fold de-repressed when AhrC was absent. Furthermore, the transcriptional reporter fusion was no longer responsive to the presence of L-arginine in the growth medium (Figures 4B,C). This transcriptional profile of the *argC-treA* reporter fusion reflects the pattern reported in previous studies on the transcriptional control of the *argCJBD-carAB-argF* operon (Smith et al., 1989; Czaplewski et al., 1992; Garnett et al., 2008).

The transcriptional profile of the six studied P*argC-treA* reporter fusions carrying *argC* operator mutations (Figure 2) was strikingly different from that of the wild-type strain. In the absence of AhrC, all strains expressed the reporter fusion constitutively at a level resembling that of the wild-type reporter fusion (Figure 4B). However, when AhrC was present, all reporter fusions carrying mutant *argC* operators were expressed at a much higher level, between 54-fold and 60-fold, than the wild-type fusion in the absence of L-arginine (Figure 4C), indicating that the binding of the AhrC regulatory protein to the mutant operators was probably reduced. However, AhrC-responsiveness of the *argC* operator variants was not completely lost as the promoter activity was still reduced by the presence of L-arginine in the medium, albeit to various degrees (Figure 4C). Collectively, the data obtained with the P*argC-treA* transcriptional reporter fusion show that the *argCJBD-carAB-argF* operon is expressed at higher levels in each of the suppressor strains carrying the operator mutations compared with the wild-type strain that possesses an intact *ahrC* gene. This is true for both the absence and for the presence of AhrC effector molecule L-arginine in the growth medium (Figures 4B,C).

### Assessment of AhrC Variants on *argC* Promoter Activity

To study the influence of the seven recovered suppressor mutations located in the *ahrC* regulatory gene (Table 2) on the expression of the *argCJBD-carAB-argF* operon, we introduced a wild-type *PargC-treA* reporter fusion into both a Δ*ahrC* strain and into each of the seven suppressor strains possessing gene variants *of ahrC*. In each of the strains carrying the *ahrC* suppressor alleles, the *PargC-treA* transcriptional reporter fusion was expressed at high levels in an L-arginine non-responsive manner (Figure 4D). Consequently, all AhrC variants obtained in our suppressor screen (Table 2) are non-functional, and hence, the defects in this regulatory protein should influence L-arginine metabolism of *B. subtilis*.

### Growth of Suppressor Strains at High-Salinity

**Figure 7:**
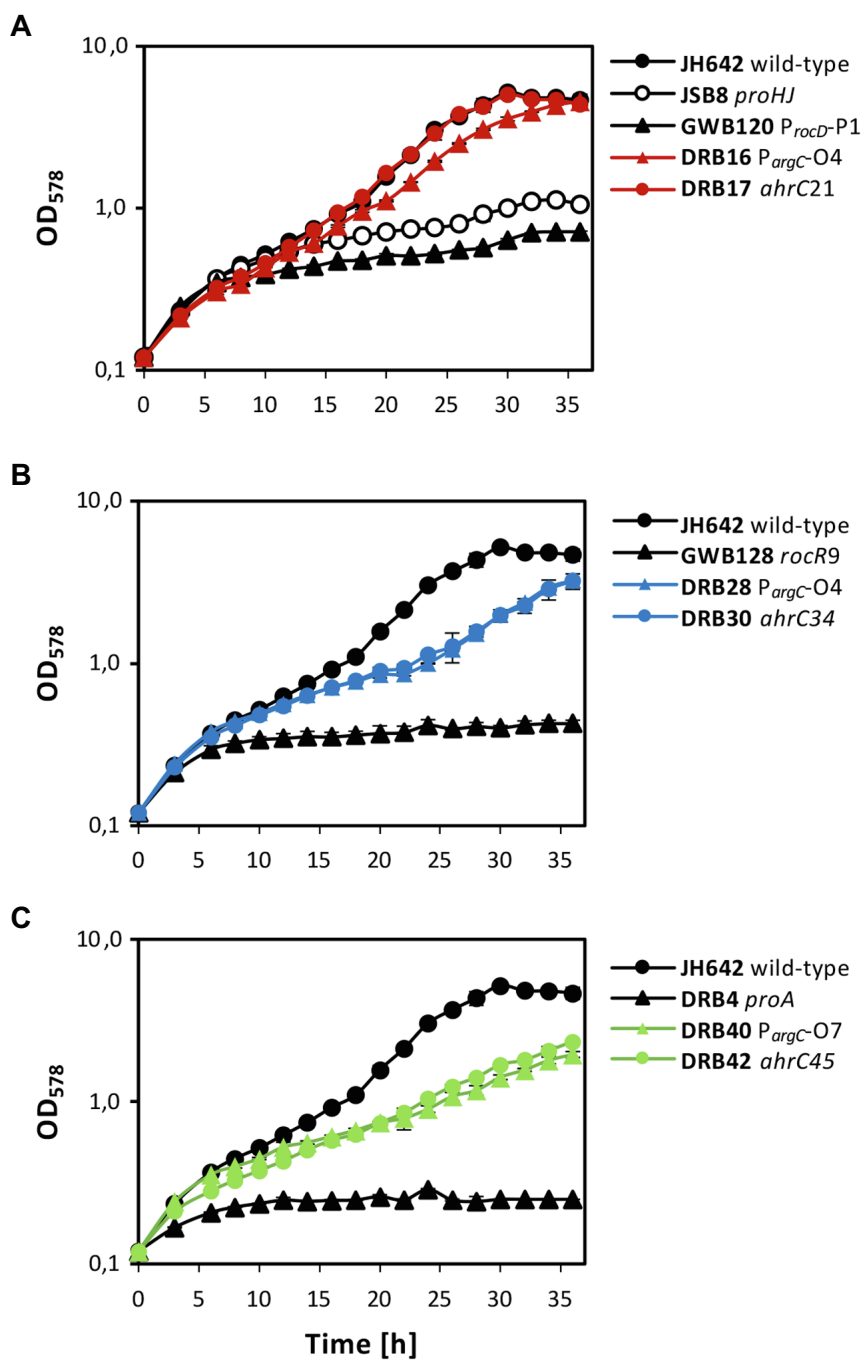
Osmostress-resistant growth phenotype of various *proA* suppressor mutants. The cells were grown in shake-flasks at 37° C in SMM containing 1.2 M NaCl. For comparison of the various suppressor strains, the growth pattern of the *B. subtilis* JH642 wild-type strain and that of a strain (JSB8) defective in the osmostress-adaptive L-proline biosynthetic route (Δ*proHJ*; Brill et al., 2011a) is shown. To aid clarity of the presentation, the same growth data documented for the wild-type strain JH642 in **(A)**, were re-used in panels **(B,C)**. In each of the panels, growth of the starting strains used for the suppressor selection (GBW120, GWB128, DRB4) and two of their suppressor derivatives is shown. The shown growth curves reflect a representative growth experiment performed with two biological replicates. These experiments were repeated in an independently conducted experiment, which yielded the same results.

After clarification of the molecular underpinnings of the types of mutations present in the collection of 27 suppressor strains isolated on SMM plates with 0.8 M NaCl (Tables 2 and 3), we chose six representatives for further physiological studies. In these strains, the original SigA promoter-up mutation for the *rocDEF* operon, or the original partial effector independent mutation in *rocR* (Zaprasis et al., 2013b) were combined with *argC* operator variants or mutations in *ahrC* (see Table 1 for the relevant genotypes of the strains; Tables 2 and 3). These six strains were grown in SMM containing 1.2 M NaCl, a severe osmotic challenge for *B. subtilis* (Boch et al., 1994). Their growth pattern was compared to that of the *B. subtilis* wild-type strain JH642, strain JSB8 (Δ*proHJ*) carrying a defect in the osmostress adaptive L-proline biosynthesis route (Brill et al., 2011a), and that of the three parent strains (DRB4 (Δ*proA*), GWB120 (Δ*proBA* P*_rocD_*-P1) and GWB128 (Δ*proBA rocR*-9; Table 1) used for the suppressor selection (Figures 3B,C). Under the imposed high salinity conditions through the presence of 1.2 M NaCl in the medium, the strain with a defective osmostress adaptive L-proline biosynthesis system (strain JSB8; Δ*proHJ*; Brill et al., 2011a), and the parent strains used for the selection of osmostress resistant suppressors barely grew. However, growth was noticeably improved for each of the six spontaneous suppressor strains newly isolated in this study (Figures 7A–C).

Both suppressor derivatives of strain GBW120 (Δ*proBA* P*_rocD_*-P1) were able to cope effectively with the high salinity growth conditions. The strain carrying a defective *ahrC* allele grew like the wild-type strain with growth rates of 0.125 h^−1^ (wild-type strain JH642) and 0.124 h^−1^ (suppressor strain DRB17), respectively. Likewise, the strain possessing the *arg*-O4 operator mutation (strain DRB16) was almost as osmotolerant as the wild-type strain JH642 and exhibited a growth rate of 0.110 h^−1^ (Figure 7A). Growth was also significantly improved in the two strains derived from strain GWB128 (Δ*proBA rocR*-9) that carried either an additional *argC* operator mutation (strain DRB28), or a mutation disrupting the function of the *ahrC* regulatory gene (strain DRB30). Yet, the growth rates of these two suppressor strains (DRB28: 0.084 h^−1^; DRB30: 0.081 h^−1^) were reduced in comparison to that exhibited by the wild-type strain JH642 (0.125 h^−1^; Figure 7B). As expected from their phenotype on minimal agar plates containing 0.8 M salt used originally for the suppressor section (Figures 3A,B), the DRB4 (Δ*proA*) derived strains DRB40 (carrying a *argC* operator mutation) and DRB42 (carrying a *ahrC* gene disruption mutation) also showed notably improved growth in comparison with their parent strain. However, the improvement of growth at a salinity of 1.2 M NaCl in liquid cultures was only moderate in comparison with that of the wild-type strain JH642 (Figure 7C). Hence, the suppressor strains in which the RocD-mediated flow of γ-glutamate-semialdehyde/Δ^1^-pyrroline-5-carboxylate into the L-proline biosynthetic route (Figure 1A) was enhanced through pre-existing mutations increasing *rocDEF* expression (Zaprasis et al., 2013b) fared best under osmotically very challenging growth conditions (Boch et al., 1994; Figure 7).

### Metabolic Changes Underpinning Osmostress Adaptation in the Suppressor Strains

We examined the metabolic changes that resulted from the suppressor mutations in the *ahrC* gene or in the *argC* operator. For this analysis we focused on the L-proline and L-arginine biosynthetic routes from their common precursor L-glutamate (Figure 1A). We analyzed the metabolites of suppressor strain DRB16, DRB17, DRB28 and DRB30 (Table 1), as these four suppressor strains showed a considerable improvement of growth at high salinity in comparison with their corresponding parent strains (Figures 7A,B).

The size of the steady-state pools of the precursor L-glutamate, key intermediates in L-arginine biosynthesis (L-ornithine and L-citrulline), and of the products L-arginine and L-proline in the absence and presence of salt stress are summarized in Figures 5A,B and in Supplementary Table S3. Consistent with previous studies (Whatmore et al., 1990; Brill et al., 2011a), the pool size of the compatible solute L-proline was about 48-fold increased (from 5 to 239 mM), while that of the L-proline biosynthetic precursor L-glutamate was concomitantly noticeably drained by about 35% (from 249 to 162 mM) when the *B. subtilis* wild-type cells were subjected to a severe and persistent salt stress (Figures 5A,B; Supplementary Table S3). The pools of the intermediates in L-arginine synthesis, L-ornithine and L-citrulline (Figure 1A) were only marginally affected in the wild-type strain JH642 by an increase in salinity. With pool sizes about 2 mM (L-ornithine) and 24 mM (L-citrulline), they were rather moderate in comparison with those of L-glutamate (126 mM) and L-proline (239 mM) pools found in the salt-stressed cells (Figures 5A,B; Supplementary Table S4).

The metabolic profile of the four studied suppressor strains differed from that of the salt-stressed *B. subtilis* wild-type strain JH642. Like the wild-type strain, each of these strains contained a substantial L-proline pool, but the most noticeable difference was an increase in the L-citrulline content of the cells. The L-citrulline pool (24 mM) was increased only by about twofold to threefold in the suppressor strains (DRB28 and DRB30) derived from strain GWB128 (Δ*proBA rocR*-9; Figure 3), but it was enhanced by about tenfold in those strains (DRB16 and DRB17) derived from strain GWB120 (Δ*proBA* P*_rocD_*-P1; Figures 5A,B; Supplementary Table S4). Each of the suppressor strains also contained substantial pools of L-glutamate, while the cellular content of L-ornithine or L-arginine were very low and comparable to that of the wild-type strain JH642 grown under persistent high salinity conditions (Figures 5A,B; Supplementary Table S4). γ-glutamate-semialdehyde, the reaction product formed either by the ProA or RocD enzymes (Figure 1A), was not detected in our analysis of the metabolome, probably because it is unstable. Large cellular pools of this metabolite are also not expected due to the high chemical reactivity and ensuing toxicity of this aldehyde.

## Discussion

Bacterial genome evolution is highly dynamic and the ability of microorganisms to finely-tune their metabolism through mutations to circumvent cellular or environmental constraints is one of the reasons for their ecological success (Barrick and Lenski, 2013). Here, we describe a series of spontaneous osmostress-tolerant suppressor strains of *B. subtilis* that overcame a *proA*-dependent genetic block of the osmostress-adaptive biosynthetic route (ProJ-ProA-ProH) for the compatible solute L-proline (Figures 1A, 3, 7). L-proline is the only compatible solute that *B. subtilis* can synthesize *de novo* (Whatmore et al., 1990), and disruption of its osmostress adaptive synthesis route causes osmotic sensitivity (Brill et al., 2011a; Hoffmann and Bremer, 2016). The suppressor strains isolated in this study achieve osmostress protection through a combination of point mutations in two different regulatory sequences, either for the *argCJBD-carAB-argF* or for the *rocDEF* operon, and in two different regulatory genes, either in *rocR* or in *ahrC*. Pairs of mutations thus upregulate the transcription of the genes for L-arginine biosynthesis and the degradation of L-ornithine, an intermediate in L-arginine synthesis, to synthesize enhanced amounts of γ-glutamate-semialdehyde/Δ^1^-pyrroline-5-carboxylate. These RocD-produced metabolites are also the products of the γ-glutamyl phosphate reductase ProA (Belitsky et al., 2001), thereby providing a metabolic by-pass for the loss of the ProA enzyme (Figure 1A).

To overcome the salt-sensitive growth phenotype of a *B. subtilis proA* mutant (Figure 7), the opening of the RocD-dependent metabolic shunt between L-ornithine catabolism and the conversion of the generated γ-glutamate-semialdehyde/Δ^1^-pyrroline-5-carboxylate by Δ^1^-pyrroline-5-carboxylate reductases is a pre-requisite to efficiently produce L-proline (Figures 5A,B; Supplementary Table S4). The suppressor strains thus make use of a naturally existing, but weakly effective (Zaprasis et al., 2013b), by-pass route interconnecting L-arginine and L-proline synthesis in *B. subtilis* (Figure 1A). The combined regulatory mutations in either of the *argCJBD-carAB-argF/rocDEF* operons and in either of the *rocR*/*ahrC* activator/repressor genes make this metabolic by-pass route more efficient, thereby resulting in the enhanced production of L-proline (Figures 5A,B; Supplementary Table S4). As *B. subtilis* repurposes this route physiologically to attain osmostress tolerance (Figures 7A,B), one can view the corresponding metabolic shunt as a form of underground metabolism (Rosenberg and Commichau, 2019). However, in contrast to a traditional view on underground metabolism (Copley, 2020; Cotton et al., 2020; Tawfik, 2020), no enzyme activities had to be evolved to produce substantial amounts of the osmostress protectant L-proline (Figures 5A,B; Supplementary Table S4) *via* the L-arginine—L-ornithine—γ-glutamate-semialdehyde/Δ^1^-pyrroline-5-carboxylate metabolic shunt.

In studies addressing the cellular adjustment of *B. subtilis* to potassium limitation (0.5 mM K^+^ present in the growth medium), suppressor mutants similar to those reported here were recovered (Gundlach et al., 2017a). Two mutations occurred in the AhrC operator of the *argCJBD-carAB-argF* operon (one of which is actually identical to suppressor P*argC*-O8; Figure 2), and one mutation was found that causes an amino acid substitution (Q22/R) in the AhrC regulatory protein (Gundlach et al., 2017a). The authors of this study concluded that increased pools of the positively charged amino acids L-ornithine, L-citrulline, and L-arginine resulting from these mutations might functionally substitute, at least to some extent, for the crucial role played by potassium for the general physiology of microbial cells (Danchin and Nikel, 2019; Korolev, 2021).

The size of potassium pools plays an important role both during the initial and the sustained cellular adjustment of *B. subtilis* to high osmolarity surroundings (Whatmore et al., 1990; Whatmore and Reed, 1990; Holtmann et al., 2003; Hoffmann and Bremer, 2016). However, we consider it unlikely that the physiological consequences of the suppressors that we isolated in the *ahrC* operator of the *argCJBD-carAB-argF* operon and in the *ahrC* gene are somehow related to potassium limitations of osmotically stressed cells. The minimal medium used in our study contains 205 mM potassium (Harwood and Archibald, 1990). Furthermore, the isolated suppressor strains are derived from the *B. subtilis* laboratory strain JH642 (Smith et al., 2014), which is proficient in both the high- and the low-affinity potassium import systems KtrAB and KtrCD, respectively (Holtmann et al., 2003; Gundlach et al., 2017b).

The four suppressor strains that we studied in greater detail, both with respect to their growth under high-salinity conditions (Figures 7A,B) and their metabolome, contained substantial amounts of L-proline, as observed for the *B. subtilis* wild-type strain (Figures 5A,B; Supplementary Table S4), despite the fact that their natural osmostress-responsive L-proline biosynthetic route is not intact (Figure 1A). The amassing of L-proline could have been guessed from the way the suppressor screen was set-up (Brill et al., 2011a; Zaprasis et al., 2013b). However, the suppressor mutants also contained, unexpectantly, substantial amounts of L-citrulline (Figures 5A,B; Supplementary Table S4). We note, that similar to the suppressors that we have isolated, those found by Gundlach et al. possessed only moderately increased pools of L-ornithine and L-arginine, while the pool of L-citrulline was substantial increased (Gundlach et al., 2017a).

The suppressor strains derived from strain GWB120 and those derived from strain GWB128 contained different amounts of L-citrulline (Figures 5A,B; Supplementary Table S4). This difference might stem from the fact that the mutation in the AhrC operator of the *argCJBD-carAB-argF* operon will only affect the expression of this particular gene cluster. In contrast, mutations in the *ahrC* regulator gene will de-repress the transcription of the *argCJBD-carAB-argF* and *argGH* operons as AhrC serves as a repressor for both gene clusters (Figure 1B; Czaplewski et al., 1992). However, mutations in *ahrC* will simultaneously also affect the level of *rocDEF* transcription because AhrC functions as an activator for this operon (Gardan et al., 1995; Klingel et al., 1995). Furthermore, the RocR-9 variant (L250/H) used in some of our strains introduces another level of complexity into this regulatory network as L-proline can serve as an effector of the mutant RocR-9 protein, a feature apparently not shared by the wild-type RocR protein (Zaprasis et al., 2013b). Hence, intricacies in the regulatory mechanisms and the ensuing substantial changes in the cellular pool size of potential effector molecules for RocR and AhrC probably provide the backdrop for the different compatible solute pools produced in the various suppressor strains. While the molecular events underpinning the different pools sizes of L-proline and L-citrulline under osmotically challenging conditions are not entirely resolved, our data collectively suggest that the substantial L-citrulline pools present in some of our suppressor strains (Figures 5A,B; Supplementary Table S4) should contribute to the balancing of turgor in *B. subtilis* (Whatmore et al., 1990; Whatmore and Reed, 1990).

There seems to be a difference in the way L-citrulline contributes to osmostress tolerance of *B. subtilis* when it is generated intracellularly through a metabolic process as observed in our suppressor strains (Figures 5A,B; Supplementary Table S4), and when it is added to the growth medium of osmotically stressed cells (Zaprasis et al., 2015). In the latter case, L-citrulline is imported and is enzymatically converted to L-proline and no osmostress-protective L-citrulline pools can seemingly be formed as the osmostress-relieving attributes of externally added L-citrulline depend entirely on an intact *proHJ* operon (Zaprasis et al., 2015). Although not explicitly tested, there were apparently no citrulline pools sufficiently high to confer osmostress tolerance in the absence of the osmostress adaptative L-proline biosynthetic route. Hence, the apparent physiological difference in the use of externally provided and internally synthesized citrulline as an osmostress protectant for *B. subtilis* warrants further study.

L-citrulline is rarely used in bacteria as a compatible solute (Da Costa et al., 1998; He et al., 2017; Chun et al., 2019) and has not been previously detected as a newly synthesized osmostress protectant in Bacilli (Kuhlmann and Bremer, 2002; Bursy et al., 2007). However, it possesses, like L-proline, chemical chaperone activity (Held et al., 2010; Choudhary et al., 2015; Held and Sadowski, 2016). This raises the question why nature has not chosen L-citrulline as a frequently used compatible solute by microorganisms (Da Costa et al., 1998; Kempf and Bremer, 1998). In contrast to L-proline that possesses a net-neutral charge at physiological pH, L-citrulline is positively charged. Its high-level accumulation might thus disturb the cells attempt to avoid a long-lasting high ionic strength cytoplasm under high osmolarity growth conditions (Wood, 2011; Van den Berg et al., 2017; Bremer and Krämer, 2019). In addition, the solubility of L-citrulline (200 g L^−1^; =1.14 M) at 20° C in water is much lower than that of L-proline (1,500 g L^−1^; =13 M), a key physico-chemical determinant for most compatible solutes used by microorganisms (Da Costa et al., 1998; Held et al., 2010; Held and Sadowski, 2016). Nevertheless, we note in this context that enhanced synthesis of L-citrulline occurs under osmotic and drought-stress in various plants where this non-proteogenic amino acid might not only serve as a protectant against water stress but also functions as a hydroxyl radical scavenger (Kawasaki et al., 2000; Akashi et al., 2001; Joshi and Fernie, 2017; Song et al., 2020). Such functions have also been suggested for newly synthesized L-citrulline in the moderate halophilic lactic acid bacterium *Tetragenococcus halophilus* exposed on a sustained basis to high-salinity environments (He et al., 2017; Chun et al., 2019).

## Data Availability Statement

The original contributions presented in the study are included in the article/Supplementary Material, further inquiries can be directed to the corresponding author.

## Author Contributions

EB conceived and supervised this study. DS conducted the physiological and genetic experiments. HL and TH performed metabolomic analysis. FC conducted the analysis of the re-sequenced genomes of various suppressor strains. TH designed all figures. EB, TH, and FC wrote the manuscript. All authors contributed to the article and approved the submitted version.

## Funding

Financial support for this work was provided to EB by the Deutsche Forschungsgemeinschaft (DFG) through the Collaborative Research Council CRC 987 (SFB 987), the LOEWE Program of the State of Hessen *via* the SNMIKRO Research Center (University of Marburg; to EB and HL), the Max Planck Society (to HL), and by the DFG (to FC).

## Conflict of Interest

The authors declare that the research was conducted in the absence of any commercial or financial relationships that could be construed as a potential conflict of interest.

## Publisher’s Note

All claims expressed in this article are solely those of the authors and do not necessarily represent those of their affiliated organizations, or those of the publisher, the editors and the reviewers. Any product that may be evaluated in this article, or claim that may be made by its manufacturer, is not guaranteed or endorsed by the publisher.

## Footnotes

^1^ https://www.graphpad.com
