# Supplementary material for: L-Proline Synthesis Mutants of Bacillus subtilis Overcome Osmotic Sensitivity by Genetically Adapting L-Arginine Metabolism
Source: Front Microbiol. 2022 Jun 16;13:908304. doi: 10.3389/fmicb.2022.908304 (PMC9245794; doi:10.3389/fmicb.2022.908304)
Supplement: Supplementary file 1 [file Data_Sheet_1.PDF]

L-proline synthesis mutants of *Bacillus subtilis* overcome osmotic sensitivity by genetically adapting L-arginine metabolism

Daniela Stecker[1,¶], Tamara Hoffmann[1,2,¶,†], Hannes Link[2,3,#,†], Fabian M. Commichau[4,5, ##,†], and Erhard Bremer[1,2]*

[1]Faculty of Biology, Philipps-University Marburg, Marburg, Germany
[2]SYNMIKRO Research Center, Philipps-University Marburg, Marburg, Germany
[3]Max Planck, Institute for Terrestrial Microbiology, Marburg, Germany
[4]Insitute of Microbiology and Genetics, Georg-August-University Göttingen, Göttingen, Germany
[5]Institute for Biotechnology, BTU Cottbus-Senftenberg, Senftenberg; Germany

[¶]Both authors contributed equally

Correspondence:

Erhard Bremer
bremer@staff.uni-marburg.de

Present Address:

[#]Dr. Hannes Link
University Tübingen
Cluster of Excellence CMFI – Bacterial Metabolomics
Auf der Morgenstelle 24
D-72076 Tübingen
Germany

[##]Dr. Fabian M. Commichau
University Hohenheim
Institute for Biology
Molecular Microbiology
Garbenstrasse 30
D-70599 Stuttgart
Germany

Running title: Attaining osmostress tolerance

For correspondence during the reviewing and editorial process please contact:

Erhard Bremer, Philipps-University Marburg, SYNMIKRO Research Center, Karl-von-Frisch Strasse 14, D-35043, Marburg, Germany. Phone: (+49)-6421-2821529; Fax: (+49)-6421-2822229; E-Mail: bremer@staff.uni-marburg.de

Table S1. *B. subtilis* strains used in this study

Strain	Relevant genotype	Suppressor name	Osmotics stress tolerance	Reference/Origin
JH642	*pheA*1 *trpC*2		+	BGSC 1A96 [a)]
JSB8	JH642 Δ(*proHJ*::*tet*)1		-	Brill *et al.*, 2011
GWB120	JH642 Δ(*proBA*::*cat*)2 (P_{rocD}-P1)	Pro^+-1	-	Zaprasis *et al.*, 2013
DRB16	JH642 Δ(*proBA*::*cat*)2 (P_{rocD}-P1) P_{argC}-O4	Pro^+-20	+	This study
DRB17	JH642 Δ(*proBA*::*cat*)2 (P_{rocD}-P1) *ahrC*21	Pro^+-21	+	This study
DRB18	JH642 Δ(*proBA*::*cat*)2 (P_{rocD}-P1) P_{argC}-O1	Pro^+-22	+	This study
DRB19	JH642 Δ(*proBA*::*cat*)2 (P_{rocD}-P1) P_{argC}-O2	Pro^+-23	+	This study
DRB20	JH642 Δ(*proBA*::*cat*)2 (P_{rocD}-P1) P_{argC}-O4	Pro^+-24	+	This study
DRB21	JH642 Δ(*proBA*::*cat*)2 (P_{rocD}-P1) P_{argC}-O4	Pro^+-25	+	This study
DRB22	JH642 Δ(*proBA*::*cat*)2 (P_{rocD}-P1) P_{argC}-O3	Pro^+-26	+	This study
DRB23	JH642 Δ(*proBA*::*cat*)2 (P_{rocD}-P1) P_{argC}-O3	Pro^+-27	+	This study
DRB24	JH642 Δ(*proBA*::*cat*)2 (P_{rocD}-P1) P_{argC}-O3	Pro^+-28	+	This study
DRB25	JH642 Δ(*proBA*::*cat*)2 (P_{rocD}-P1) P_{argC}-O3	Pro^+-29	+	This study
DRB26	JH642 Δ(*proBA*::*cat*)2 (P_{rocD}-P1) P_{argC}-O3	Pro^+-30	+	This study
DRB27	JH642 Δ(*proBA*::*cat*)2 (P_{rocD}-P1) P_{argC}-O3	Pro^+-31	+	This study
GWB128	JH642 Δ(proBA::*cat*)2 *rocR*9	Pro^+-9	-	Zaprasis *et al.*, 2013
DRB28	JH642 Δ(proBA::*cat*)2 *rocR*9 P_{argC}-O4	Pro^+-32	+	This study
DRB30	JH642 Δ(proBA::*cat*)2 *rocR*9 *ahrC*34	Pro^+-34	+	This study
DRB31	JH642 Δ(*proBA*::*cat*)2 (P_{rocD}-P1) *ahrC*35	Pro^+-35	+	This study
DRB33	JH642 Δ(*proBA*::*cat*)2 (P_{rocD}-P1) *ahrC*36	Pro^+-36	+	This study
DRB34	JH642 Δ(proBA::*cat*)2 *rocR*9 P_{argC}-O6	Pro^+-37	+	This study
DRB35	JH642 Δ(proBA::*cat*)2 *rocR*9 P_{argC}-O6	Pro^+-38	+	This study
DRB36	JH642 Δ(proBA::*cat*)2 *rocR*9 P_{argC}-O5	Pro^+-39	+	This study
DRB37	JH642 Δ(proBA::*cat*)2 *rocR*9 P_{argC}-O2	Pro^+-40	+	This study
DRB38	JH642 Δ(proBA::*cat*)2 *rocR*9 P_{argC}-O2	Pro^+-41	+	This study
DRB39	JH642 Δ(*proBA*::*cat*)2 (P_{rocD}-P1) *ahrC*42	Pro^+-42	+	This study
DRB4	JH642 Δ(proA::ery)		-	This study
DRB40	JH642 Δ(proA::ery) P_{argC}-O7	Pro^+-43	+	This study
DRB41	JH642 Δ(proA::ery) P_{argC}-O8	Pro^+-44	+	This study
DRB42	JH642 Δ(proA::ery) *ahrC*45	Pro^+-45	+	This study
DRB43	JH642 Δ(proA::ery) *ahrC*46	Pro^+-46	+	This study

[a)] BGSC: Bacillus Genetic Stock Center (Columbia, OH, USA)

**Strain**	**Relevant genotype**	**Reference/ Origin**
DRB62	JH642 Δ(*ahrC*::*ery*)1 Δ(*treA*::*kan*)1 *amyE*::ϕ(P_{argC}-O1-*treA*, *cat*)	This study
DRB63	JH642 Δ(*ahrC*::*ery*)1 Δ(*treA*::*kan*)1 *amyE*::ϕ(P_{argC}-O3-*treA*, *cat*)	This study
DRB64	JH642 Δ(*ahrC*::*ery*)1 Δ(*treA*::*kan*)1 *amyE*::ϕ(P_{argC}-O8-*treA*, *cat*)	This study
DRB65	JH642 Δ(*ahrC*::*ery*)1 Δ(*treA*::*kan*)1 *amyE*::ϕ(P_{argC}-O4-*treA*, *cat*)	This study
DRB66	JH642 Δ(*ahrC*::*ery*)1 Δ(*treA*::*kan*)1 *amyE*::ϕ(P_{argC}-O5-*treA*, *cat*)	This study
DRB67	JH642 Δ(*ahrC*::*ery*)1 Δ(*treA*::*kan*)1 *amyE*::ϕ(P_{argC}-O6-*treA*, *cat*)	This study
DRB68	JH642 Δ(*ahrC*::*ery*)1 Δ(*treA*::*kan*)1 *amyE*::ϕ(P_{argC}-wild type-*treA*, *cat*)	This study
DRB69	JH642 Δ(*proBA*::*cat*)2 (P_{rocD}-P1) *ahrC*21 Δ(*treA*::*kan*)1 *amyE*::ϕ(P_{argC}-*treA*, *cat*)	This study
DRB70	JH642 Δ(*proBA*::*cat*)2 *rocR*9 *ahrC*34 Δ(*treA*::*kan*)1 *amyE*::ϕ(P_{argC}-*treA*, *cat*)	This study
DRB71	JH642 Δ(*proBA*::*cat*)2 *rocR*9 *ahrC*35 Δ(*treA*::*kan*)1 *amyE*::ϕ(P_{argC}-*treA*, *cat*)	This study
DRB72	JH642 Δ(*proBA*::*cat*)2 *rocR*9 *ahrC*36 Δ(*treA*::*kan*)1 *amyE*::ϕ(P_{argC}-*treA*, *cat*)	This study
DRB73	JH642 Δ(*proBA*::*cat*)2 *rocR*9 *ahrC*42 Δ(*treA*::*kan*)1 *amyE*::ϕ(P_{argC}-*treA*, *cat*)	This study
DRB74	JH642 Δ(*proA*::*ery*) *ahrC*45 Δ(*treA*::*kan*)1 *amyE*::ϕ(P_{argC}-*treA*, *cat*)	This study
DRB75	JH642 Δ(*proA*::*ery*) *ahrC*46 Δ(*treA*::*kan*)1 *amyE*::ϕ(P_{argC}-*treA*, *cat*)	This study
DRB76	JH642 Δ(*treA*::*ery*)2 *amyE*::ϕ(P_{argC}-O1-*treA*, *cat*)	This study
DRB77	JH642 Δ(*treA*::*ery*)2 *amyE*::ϕ(P_{argC}-O3-*treA*, *cat*)	This study
DRB78	JH642 Δ(*treA*::*ery*)2 *amyE*::ϕ(P_{argC}-O4-*treA*, *cat*)	This study
DRB79	JH642 Δ(*treA*::*ery*)2 *amyE*::ϕ(P_{argC}-O5-*treA*, *cat*)	This study
DRB80	JH642 Δ(*treA*::*ery*)2 *amyE*::ϕ(P_{argC}-O6-*treA*, *cat*)	This study
DRB81	JH642 Δ(*treA*::*ery*)2 *amyE*::ϕ(P_{argC}-O8-*treA*, *cat*)	This study
DRB82	JH642 Δ(*treA*::*ery*)2 *amyE*::ϕ(P_{argC}-wild type-*treA*, *cat*)	This study

Table S2. Plasmids used in this study

Plasmid	Description	Reference/Origin
pPink1	low-copy number *treA* operon fusion vector; derivative of pJMB1, contains a two base pair insertion between the *Sma*I and *Bam*HI restriction sites which allows more efficient cloning	Hoffmann *et al.,* 2011
pDST32	pPink1 derivative; with P*argC*-O1 fragment fused to *treA*	This study
pDST34	pPink1 derivative; with P*argC*-O3 fragment fused to *treA*	This study
pDST35	pPink1 derivative; with P*argC*-O4 fragment fused to *treA*	This study
pDST36	pPink1 derivative; with P*argC*-O5 fragment fused to *treA*	This study
pDST37	pPink1 derivative; with P*argC*-O6 fragment fused to *treA*	This study
pDST39	pPink1 derivative; with P*argC*-O8 fragment fused to *treA*	This study
pDST40	pPink1 derivative; with P*argC*-wild type fragment fused to *treA*	This study

Table S3. Significance values (P values) of data shown in Figure 5

Data analyzed	Figure 5 B without Arginine						
Strain 1	P_{argC}-O1	P_{argC}-O3	P_{argC}-O4	P_{argC}-O5	P_{argC}-O6	P_{argC}-O8	
vs.	vs,	vs,	vs,	vs,	vs,	vs,	
Strain 2	P_{argC}-wt	P_{argC}-wt	P_{argC}-wt	P_{argC}-wt	P_{argC}-wt	P_{argC}-wt	
Unpaired test [a]							
P value	0,0324	0,1132	0,0107	0,7783	0,0503	0,012	
P value summary	*	ns	*	ns	ns	*	
Significantly different (P < 0.05)?	Yes	No	Yes	No	No	Yes	
**Data analyzed**	**Figure 5 B 20 mM Arginine**						
Strain 1	P_{argC}-O1	P_{argC}-O3	P_{argC}-O4	P_{argC}-O5	P_{argC}-O6	P_{argC}-O8	
vs.	vs,	vs,	vs,	vs,	vs,	vs,	
Strain 2	P_{argC}-wt	P_{argC}-wt	P_{argC}-wt	P_{argC}-wt	P_{argC}-wt	P_{argC}-wt	
Unpaired test [a]							
P value	0,0179	0,0335	0,0009	0,1984	0,004	0,0012	
P value summary	*	*	***	ns	**	**	
Significantly different (P < 0.05)?	Yes	Yes	Yes	No	Yes	Yes	
**Data analyzed**	**Figure 5 C without Arginine**						
Strain 1	P_{argC}-O1	P_{argC}-O3	P_{argC}-O4	P_{argC}-O5	P_{argC}-O6	P_{argC}-O8	
vs.	vs,	vs,	vs,	vs,	vs,	vs,	
Strain 2	P_{argC}-wt	P_{argC}-wt	P_{argC}-wt	P_{argC}-wt	P_{argC}-wt	P_{argC}-wt	
Unpaired test							
P value	0,0017	0,0004	<0,0001	0,0001	<0,0001	0,0006	
P value summary	**	***	****	***	****	***	
Significantly different (P < 0.05)?	Yes	Yes	Yes	Yes	Yes	Yes	
**Data analyzed**	**Figure 5 C 20 mM Arginine**						
Strain 1	P_{argC}-O1	P_{argC}-O3	P_{argC}-O4	P_{argC}-O5	P_{argC}-O6	P_{argC}-O8	
vs.	vs,	vs,	vs,	vs,	vs,	vs,	
Strain 2	P_{argC}-wt	P_{argC}-wt	P_{argC}-wt	P_{argC}-wt	P_{argC}-wt	P_{argC}-wt	
Unpaired test							
P value	0,0004	0,0009	0,0015	0,0015	0,0004	<0,0001	
P value summary	***	***	**	**	***	****	
Significantly different (P < 0.05)?	Yes	Yes	Yes	Yes	Yes	Yes	
**Data analyzed**	**Figure 5 D without Arginine**						
Strain 1	DRB69 AhrC21	DRB70 $AhrC^{T40K}$	DRB71 $AhrC^{Q38A}$	DRB72 $AhrC^{G101D}$	DRB73 AhrC42	DRB74 AhrC45	DRB75 $AhrC^{A103D}$
vs.	vs,	vs,	vs,	vs,	vs,	vs,	vs,
Strain 2	DRB68 Δ*ahrC*	DRB68 Δ*ahrC*	DRB68 Δ*ahrC*	DRB68 Δ*ahrC*	DRB68 Δ*ahrC*	DRB68 Δ*ahrC*	DRB68 Δ*ahrC*
Unpaired test							
P value	0,1498	0,0822	0,0172	0,054	0,0039	0,8084	0,0036
P value summary	ns	ns	*	ns	**	ns	**
Significantly different (P < 0.05)?	No	No	Yes	No	Yes	No	Yes
**Data analyzed**	**Figure 5 D 20 mM Arginine**						
Strain 1	DRB69 AhrC21	DRB70 $AhrC^{T40K}$	DRB71 $AhrC^{Q38A}$	DRB72 $AhrC^{G101D}$	DRB73 AhrC42	DRB74 AhrC45	DRB75 $AhrC^{A103D}$
vs.	vs,	vs,	vs,	vs,	vs,	vs,	vs,
Strain 2	DRB68 Δ*ahrC*	DRB68 Δ*ahrC*	DRB68 Δ*ahrC*	DRB68 Δ*ahrC*	DRB68 Δ*ahrC*	DRB68 Δ*ahrC*	DRB68 Δ*ahrC*
Unpaired test							
P value	0,3865	0,4114	0,5257	0,8856	0,5508	0,4971	0,7644
P value summary	ns	ns	ns	ns	ns	ns	ns
Significantly different (P < 0.05)?	No	No	No	No	No	No	No

[a] P values were calculated using the "unpaired t test" analysis tool in Prism 9 (GraphPad Software, San Diego, CA, USA). ns: not significant

Table S4. Intracellular metabolite concentrations of selected Pro$^+$-suppressor strains

Suppressor name	NaCl [M]	L-Glu [mM]	L-Pro [mM]	L-Cit [mM]	L-Orn [mM]	L-Arg [mM]	total [mM]
wild type	0	249 ± 13	5 ± 1	11 ± 2	0,7 ± 0.3	0.4 ± 0.3	**266**
	1.2	162 ± 42	239 ± 28	24 ± 3	1,9 ± 2.1	0.2 ± 0.0	**428**
GWB120 (P_{rocD}-P1)-derived							
DRB16 (P_{argC}-O4)	0	119 ± 18	8 ± 1	204 ± 16	1,1 ± 0.3	0.4 ± 0.2	**332**
	1.2	148 ± 64	103 ± 29	244 ± 62	3.2 ± 3.8	0.3 ± 0.1	**498**
DRB17 (*ahrC*21)	0	139 ± 39	14 ± 4	85 ± 27	3.6 ± 1.2	1.8 ± 0.3	**243**
	1.2	144 ± 35	145 ± 60	237 ± 28	3.6 ± 3.0	2.5 ± 0.5	**531**
GWB128 (*rocR*9)-derived							
DRB28 (P_{argC}-O4)	0	165 ± 23	19 ± 3	38 ± 8	2.2 ± 0.2	0.3 ± 0.1	**225**
	1.2	98 ± 5	185 ± 78	74 ± 9	0.7 ± 0.2	0.3 ± 0.1	**357**
DRB30 (*ahrC*34)	0	224 ± 25	15 ± 6	17 ± 2	4.2 ± 0.7	0.7 ± 0.1	**261**
	1.2	122 ± 31	207 ± 34	55 ± 23	0.6 ± 0.1	0.4 ± 0.1	**385**

[a] Metabolites from the *B. subtilis* strain JH642 and the suppressor mutants were analyzed from cells that had been grown in SMM (no additional NaCl) and in SMM with additional 1.2 M NaCl. Cells were harvested in the early-exponential growth phase (OD_{578} of 1.5). For growth curves of the corresponding strains see Figure 6

[b] Sum of all listed solutes. Individual standard deviations are given with the solutes, respectively.

Table S5. Significance values (P values) of metabolome data shown in Table S4 and Figure 7

Metabolite analyzed	**L-Glutamate**	**L-Glutamate**	**L-Glutamate**	**L-Glutamate**	**L-Glutamate**	**L-Glutamate**	**L-Glutamate**	**L-Glutamate**
Strain 1	DRB16	DRB17	DRB28	DRB30	DRB16	DRB17	DRB28	DRB30
	no NaCl	no NaCl	no NaCl	no NaCl	1,2 M NaCl	1,2 M NaCl	1,2 M NaCl	1,2 M NaCl
	vs,	vs,	vs,	vs,	vs,	vs,	vs,	vs,
Strain 2	JH642	JH642	JH642	JH642	JH642	JH642	JH642	JH642
	no NaCl	no NaCl	no NaCl	no NaCl	1,2 M NaCl	1,2 M NaCl	1,2 M NaCl	1,2 M NaCl
Unpaired t test [a)]								
P value	<0,0001	0,0017	0,0007	0,1265	0,7373	0,5281	0,1097	0,1761
P value summary	****	**	***	ns	ns	ns	ns	ns
Significantly different (P < 0.05)?	Yes	Yes	Yes	No	No	No	No	No

Metabolite analyzed	**L-Proline**	**L-Proline**	**L-Proline**	**L-Proline**	**L-Proline**	**L-Proline**	**L-Proline**	**L-Proline**
Strain 1	DRB16	DRB17	DRB28	DRB30	DRB16	DRB17	DRB28	DRB30
	no NaCl	no NaCl	no NaCl	no NaCl	1,2 M NaCl	1,2 M NaCl	1,2 M NaCl	1,2 M NaCl
	vs,	vs,	vs,	vs,	vs,	vs,	vs,	vs,
Strain 2	JH642	JH642	JH642	JH642	JH642	JH642	JH642	JH642
	no NaCl	no NaCl	no NaCl	no NaCl	1,2 M NaCl	1,2 M NaCl	1,2 M NaCl	1,2 M NaCl
Unpaired t test								
P value	0,0003	0,0039	<0,0001	0,0138	0,0005	0,029	0,2353	0,2292
P value summary	***	**	****	*	***	*	ns	ns
Significantly different (P < 0.05)?	Yes	Yes	Yes	Yes	Yes	Yes	No	No

Metabolite analyzed	**L-Citrulline**	**L-Citrulline**	**L-Citrulline**	**L-Citrulline**	**L-Citrulline**	**L-Citrulline**	**L-Citrulline**	**L-Citrulline**
Strain 1	DRB16	DRB17	DRB28	DRB30	DRB16	DRB17	DRB28	DRB30
	no NaCl	no NaCl	no NaCl	no NaCl	1,2 M NaCl	1,2 M NaCl	1,2 M NaCl	1,2 M NaCl
	vs,	vs,	vs,	vs,	vs,	vs,	vs,	vs,
Strain 2	JH642	JH642	JH642	JH642	JH642	JH642	JH642	JH642
	no NaCl	no NaCl	no NaCl	no NaCl	1,2 M NaCl	1,2 M NaCl	1,2 M NaCl	1,2 M NaCl
Unpaired t test								
P value	<0,0001	0,0001	<0,0001	0,003	<0,0001	<0,0001	<0,0001	0,0125
P value summary	****	***	****	**	****	****	****	*
Significantly different (P < 0.05)?	Yes	Yes	Yes	Yes	Yes	Yes	Yes	Yes

Metabolite analyzed	**L-Ornithin**	**L-Ornithin**	**L-Ornithin**	**L-Ornithin**	**L-Ornithin**	**L-Ornithin**	**L-Ornithin**	**L-Ornithin**
Strain 1	DRB16	DRB17	DRB28	DRB30	DRB16	DRB17	DRB28	DRB30
	no NaCl	no NaCl	no NaCl	no NaCl	1,2 M NaCl	1,2 M NaCl	1,2 M NaCl	1,2 M NaCl
	vs,	vs,	vs,	vs,	vs,	vs,	vs,	vs,
Strain 2	JH642	JH642	JH642	JH642	JH642	JH642	JH642	JH642
	no NaCl	no NaCl	no NaCl	no NaCl	1,2 M NaCl	1,2 M NaCl	1,2 M NaCl	1,2 M NaCl
Unpaired t test								
P value	0,0761	0,0029	0,0001	<0,0001	0,5713	0,3989	0,4729	0,4671
P value summary	ns	**	***	****	ns	ns	ns	ns
Significantly different (P < 0.05)?	No	Yes	Yes	Yes	No	No	No	No

Metabolite analyzed	**L-Arginine**	**L-Arginine**	**L-Arginine**	**L-Arginine**	**L-Arginine**	**L-Arginine**	**L-Arginine**	**L-Arginine**
Strain 1	DRB16	DRB17	DRB28	DRB30	DRB16	DRB17	DRB28	DRB30
	no NaCl	no NaCl	no NaCl	no NaCl	1,2 M NaCl	1,2 M NaCl	1,2 M NaCl	1,2 M NaCl
	vs,	vs,	vs,	vs,	vs,	vs,	vs,	vs,
Strain 2	JH642	JH642	JH642	JH642	JH642	JH642	JH642	JH642
	no NaCl	no NaCl	no NaCl	no NaCl	1,2 M NaCl	1,2 M NaCl	1,2 M NaCl	1,2 M NaCl
Unpaired t test								
P value	0,6357	0,0004	0,3075	0,0747	0,71	0,0226	0,71	0,1296
P value summary	ns	***	ns	ns	ns	*	ns	ns
Significantly different (P < 0.05)?	No	Yes	No	No	No	Yes	No	No

[a)] P values were calculated using the "unpaired t test" analysis tool in Prism 9 (GraphPad Software, San Diego, CA, USA). ns: not significant

REFERENCES

Brill, J., Hoffmann, T., Bleisteiner, M., and Bremer, E. (2011) Osmotically controlled synthesis of the compatible solute proline is critical for cellular defense of *Bacillus subtilis* against high osmolarity. *J. Bacteriol.* **193,** 5335-5346.

Hoffmann, T., and Bremer, E. (2011) Protection of *Bacillus subtilis* against cold stress via compatible-solute acquisition. *J. Bacteriol.* 193, 1552-1562.

Zaprasis, A., Hoffmann, T., Wünsche, G., Florez, L.A., Stülke, J., and Bremer, E. (2013) Mutational activation of the RocR activator and of a cryptic *rocDEF* promoter bypass loss of the initial steps of proline biosynthesis in *Bacillus subtilis*. *Env. Microbiol.* 16, 701-717.
